# Utilization of low-molecular-weight organic compounds by the filterable fraction of a lotic microbiome

**DOI:** 10.1093/femsec/fiaa244

**Published:** 2020-12-02

**Authors:** Lydia-Ann J Ghuneim, Marco A Distaso, Tatyana N Chernikova, Rafael Bargiela, Evgenii A Lunev, Aleksei A Korzhenkov, Stepan V Toshchakov, David Rojo, Coral Barbas, Manuel Ferrer, Olga V Golyshina, Peter N Golyshin, David L Jones

**Affiliations:** School of Natural Sciences, Bangor University, Bangor, Gwynedd, LL57 2UW, UK; School of Natural Sciences, Bangor University, Bangor, Gwynedd, LL57 2UW, UK; Centre for Environmental Biotechnology, Bangor University, Bangor, Gwynedd, LL57 2UW, UK; School of Natural Sciences, Bangor University, Bangor, Gwynedd, LL57 2UW, UK; Centre for Environmental Biotechnology, Bangor University, Bangor, Gwynedd, LL57 2UW, UK; School of Natural Sciences, Bangor University, Bangor, Gwynedd, LL57 2UW, UK; Centre for Environmental Biotechnology, Bangor University, Bangor, Gwynedd, LL57 2UW, UK; Institute of Living Systems, Immanuel Kant Baltic Federal University, Kaliningrad, Russia; Kurchatov Center for Genome Research, National Research Center “Kurchatov Institute”, Moscow, Russian Federation; Winogradsky Institute of Microbiology, FRC Biotechnology, Russian Academy of Sciences, Moscow, Russian Federation; Centro de Metabolómica y Bioanálisis (CEMBIO), Facultad de Farmacia, Universidad CEU San Pablo, Campus Montepríncipe, Madrid, Spain; Centro de Metabolómica y Bioanálisis (CEMBIO), Facultad de Farmacia, Universidad CEU San Pablo, Campus Montepríncipe, Madrid, Spain; Institute of Catalysis, Consejo Superior de Investigaciones Científicas (CSIC), Madrid, Spain; School of Natural Sciences, Bangor University, Bangor, Gwynedd, LL57 2UW, UK; Centre for Environmental Biotechnology, Bangor University, Bangor, Gwynedd, LL57 2UW, UK; School of Natural Sciences, Bangor University, Bangor, Gwynedd, LL57 2UW, UK; Centre for Environmental Biotechnology, Bangor University, Bangor, Gwynedd, LL57 2UW, UK; School of Natural Sciences, Bangor University, Bangor, Gwynedd, LL57 2UW, UK; UWA School of Agriculture and Environment, The University of Western Australia, Perth, WA 6009, Australia

**Keywords:** microbial ecology, dissolved organic matter (DOM), filterable microorganisms, freshwater, ^14^C-radioisotope tracking, metabolomics, 16S rRNA amplicon sequencing, shotgun sequencing

## Abstract

Filterable microorganisms participate in dissolved organic carbon (DOC) cycling in freshwater systems, however their exact functional role remains unknown. We determined the taxonomic identity and community dynamics of prokaryotic microbiomes in the 0.22 µm-filtered fraction and unfiltered freshwater from the Conwy River (North Wales, UK) in microcosms and, using targeted metabolomics and ^14^C-labelling, examined their role in the utilization of amino acids, organic acids and sugars spiked at environmentally-relevant (nanomolar) concentrations. To identify changes in community structure, we used 16S rRNA amplicon and shotgun sequencing. Unlike the unfiltered water samples where the consumption of DOC was rapid, the filtered fraction showed a 3-day lag phase before the consumption started. Analysis of functional categories of clusters of orthologous groups of proteins (COGs) showed that COGs associated with energy production increased in number in both fractions with substrate addition. The filtered fraction utilized low-molecular-weight (LMW) DOC at much slower rates than the whole community. Addition of nanomolar concentrations of LMW DOC did not measurably influence the composition of the microbial community nor the rate of consumption across all substrate types in either fraction. We conclude that due to their low activity, filterable microorganisms play a minor role in LMW DOC processing within a short residence time of lotic freshwater systems.

## Teaser

This study resolved the taxonomic makeup, functionality and contribution to carbon cycling of previously largely neglected filterable freshwater microorganisms.

## INTRODUCTION

The term ‘filterable microorganisms’ refers to (i) nano-sized microorganisms, i.e. small-bodied microorganisms that have dimensions of 50–400 nm and a volume <0.1 µm^3^, (ii) larger cells that have the capability to squeeze through filters with pore sizes of <0.45 µm, and (iii) small-cell variants of microorganisms with larger cell sizes (e.g. dormant or senescent forms) (Velimirov 2001; Panikov 2005; Duda *et al*. 2012; Ghuneim *et al*. 2018; Proctor *et al*. 2018). Our knowledge of the bacteria and archaea in the filterable fraction, however, remains limited, as most of these microorganisms have proven difficult to culture under laboratory conditions (Ghuneim *et al*. 2018). Even so, several small freshwater bacteria have previously been isolated (e.g. freshwater ultramicrobacteria from the phylum *Betaproteobacterium* (Salcher and Šimek 2016), a freshwater SAR11 (LD12 subclade) *Alphaproteobacterium* (Henson *et al*. 2018) and ubiquitous ac1 lineage of *Actinobacteria* (Kim *et al*. 2019). Although some important filterable microorganisms have already been cultivated from this ecological niche, their community functions remain poorly understood (Ghuneim *et al*. 2018). Nonetheless, filterable microorganisms appear ubiquitous throughout the biosphere and have been implicated in many geochemical processes ranging from sulfur reduction in pelagic systems to the consumption of photo-oxidation products of humic substances and the production and use of dissolved organic matter (DOM) (Salcher 2014; Dang and Lovell 2016; Ghuneim *et al*. 2018).

DOM is defined as compounds that have the ability to pass through a 0.45 µm filter and is the main source of organic nutrients in freshwater systems (Brailsford *et al*. 2017). This pool of DOM is composed of many thousands of compounds, all of which differ in their chemistry, shape, molecular weight and charge (Mostovaya *et al*. 2017). Despite this, it is expected that DOM cycling will be dominated by the microbial breakdown of monomers and oligomers released during the breakdown of biological polymers (e.g. amino acids, peptides, sugars, phenolics). Most of this DOM enters lotic systems from external sources (i.e. agricultural runoff, leaf detritus, wastewater discharges) and is subsequently consumed by heterotrophic bacteria within the water column or sediment (Sigee 2005). While low concentrations of DOM rarely represent a threat to freshwaters, high concentrations can induce hypoxia or algal blooms (Beman, Arrigo and Matson 2005). Further, levels of DOM have been steadily increasing within many freshwaters over the last 50 years in response to a range of factors (e.g. land use change, changes in atmospheric emissions, climate change) (Ritson *et al*. 2014). It is therefore important to characterise the factors regulating DOM transformation in freshwaters. The study of DOM in lotic systems, however, remains challenging due to: (i) the inherent ever-changing conditions of lotic systems (i.e. flow, weather events); (ii) the difficulty of performing DOM transformation studies *in situ*; (iii) temporal changes in DOM inputs; and (iv) difficulties in chemically characterising the DOM present (Meyer 1994; Sigee 2005; Fenchel 2008). To address this, a range of *ex situ* techniques including 16S rRNA amplicon sequencing, fluorescence *in situ* hybridization (FISH), stable isotope probing (SIP), stable isotope imaging (NanoSIMS) and radioisotope labelling have been used to study the fate of DOM (Roszak and Colwell 1987; Findlay *et al*. 2003; Kirchman *et al*. 2004; Malmstrom *et al*. 2005; Rinta-Kanto *et al*. 2012; Landa *et al*. 2013). Through these various techniques, it has been demonstrated that the heterotrophic microbial communities within aquatic systems are highly diverse and their taxonomic makeup is dependent upon the levels of DOM.

A previous study on DOM cycling in the Conwy River (UK) showed the organisms passing through a 0.22 µm filter could use a range of organic and inorganic nutrients (Brailsford *et al*. 2017). This is significant because it is normally thought that ultra-filtering aqueous samples would render the sample sterile (Brailsford *et al*. 2017; Ghuneim *et al*. 2018). It was concluded that there is a potentially robust community of filterable microorganisms which can actively participate in DOM cycling (Brailsford *et al*. 2017). The purpose of this study was to use radioisotope tracking, targeted metabolomics, 16S rRNA amplicon sequencing and shotgun sequencing to compare the taxonomic composition and functional significance of microorganisms in 0.22 µm filtered aqueous samples (filterable microorganism community) versus those in the entire community (unfiltered community). We hypothesized that the filterable microbial fraction will be taxonomically distinct, metabolically active and capable of using a range of simple ‘vital-for-life’ DOM substrates at very low (nanomolar concentrations.

## METHODS

### Description of sampling site

The Conwy catchment is located in North Wales (UK) and its main drainage is the Conwy River (Afon Conwy). Mean annual rainfall ranges from 500 to 3500 mm and the mean annual air temperature ranges from 5 to15°C with an average annual temperature of 10°C (Emmett *et al*. 2016). The river is 55 km long, starting from Llyn Conwy (450 m above sea level), and drains the Migneint, a large peatland bog that is a major store of carbon. Three tributaries (Machno, Lledr and Llugwy), originating from the eastern side of the Snowdonia mountain range, join the main river further downstream before reaching the tidal limit (20 km inland) (Emmett *et al*. 2016). Average concentrations at the tidal limit are as follows (mg/L): nitrite 0.2–2.8, ammonium <0.03–0.04,  phosphate <0.02–0.05 and dissolved organic carbon (DOC) 1.5–10 (Emmett *et al*. 2016). The pH ranges from 5.0 to 7.2. The primary site used in this study (code: NM29) is located at Cwm Llanerch and is associated with the main Conwy River (53° 6' 24.7068'' N, 3° 47' 28.7556'' W). The site is located ∼4 km from the tidal limit (Simpson, Vennell and Souza 2001).

### Materials

Stock solutions of amino acids, sugars and organic acids were generated. The amino acid stock was created by adding 600 µL of L-isomeric amino acid standard H (Thermo Fisher Scientific) (L-alanine, L-arginine, L-aspartic acid, L-cystine, L-glutamic acid, glycine, L-histidine, L-isoleucine, L-leucine, L-lysine HCL, L-methionine, L-phenylalanine, L-proline, L-serine, L-threonine, L-tyrosine, L-valine) to 100 mL of LC-MS water. Subsequent serial dilution achieved a final concentration of each amino acid of 15.05 nM per sample replicate. The sugar stock was created to achieve a final concentration of D-(+)-glucose and D-(+)-fructose of 33.4 nM each and sucrose of 31.6 nM per sample/replicate. The final concentration of each organic acid (formic, L-(-)-malic and citric acids) per sample/replicate was 33.4 nM via serial dilution. All sugars, organic acids and LC-MS water were purchased from Sigma-Aldrich with purities ≥99%.The substrate choice and concentration were chosen to reflect common low-molecular-weight (LMW) substrates found in freshwaters (Brailsford *et al*. 2017).

### Sample collection and substrate addition

Approximately 2 L of freshwater was collected from Cwm Llanerch on 6 March 2018. The collected samples were either unfiltered or filtered on-site through a 0.22 µm Sterivex^TM^ Durapore^®^ PVDF (hydrophilic polyvinylidene fluoride) filter (Millipore Corporation, Billerica, MA, USA). Samples were kept on ice and in the dark during transportation. The EC (electrical conductivity) and the pH were measured for both unfiltered (EC = 68 µS/cm, pH = 6.52) and filtered (EC = 66 µS/cm, pH = 6.48) water samples. Subsequent nutrient amendments via stock solutions were added dependent on whether the sample was being analysed using non-radiolabelled or radiolabelled assays, *vide infra*. For the radiolabelled assays, 100 mL of sample (filtered, unfiltered and blank) was placed into a sterile 250 mL screw-cap Erlenmeyer flask. Subsequently, 1 ml of ^14^C-substrate was added to the water, a 15 mL 1 M sodium hydroxide trap added and the flasks sealed (radiolabelled assay). An identical set of flasks was also set up for metabolites and metagenomic analysis but to which no ^14^C substrate was added (non-radiolabelled assays). All blank samples comprised autoclaved LC-MS-grade water. All flasks were stored at room temperature (20°C) with limited light exposure. Throughout the experiment, the flasks were subjected to light agitation via swirling during measurements and collections.

### Radiolabelled assays

^14^C Radioisotopes were used to determine: (i) the depletion of added substrate from solution, (ii) substrate-induced respiration (CO_2_ production), and (iii) incorporation of C by the microorganisms into biomass (C immobilization) in the filtered and unfiltered water samples. The filtered and unfiltered samples were spiked with one type of the radiolabelled ^14^C radioisotope (three different forms) separately, all with 0.1 kBq/mL activity: (a) radiolabelled amino acid stock solution: ^14^C-amino acid mix (PerkinElmer, MA, USA Lot 3590,279; 3.7 MBq/mL) [Alanine 0.296 MBq/mL, arginine 0.259 MBq/mL, aspartic acid 0.296 MBq/mL, glutamic acid 0.462 MBq/mL, glycine 0.148 MBq/mL, histidine 0.0555 MBq/mL, isoleucine 0.185 MBq/mL, leucine 0.518 MBq/mL, lysine 0.222 MBq/mL, phenylalanine 0.296 MBq/mL, proline 0.185 MBq/mL, serine 0.148 MBq/mL, threonine 0.185 MBq/mL, tyrosine 0.148 MBq/mL and valine 0.296 MBq/mL]; (b) radiolabelled sugar stock solution: ^14^C-glucose (PerkinElmer, MA, USA; Lot 3632,475; 7.4 MBq/mL), ^14^C-fructose (PerkinElmer, MA, USA; 3.7 MBq/mL) and ^14^C-sucrose (PerkinElmer, MA, USA; 3.7 MBq/mL); or (c) radiolabelled organic acid stock solution: ^14^C-citric acid (PerkinElmer, MA, USA; 1.85 MBq/mL), ^14^C-formic acid (PerkinElmer, MA, USA; 3.49 MBq/mL) and ^14^C-malic acid (PerkinElmer, MA, USA; 3.7 MBq/mL). The concentration per each substrate per sample (microcosm) is listed in Table S1, see online supplementary material.

For substrate depletion experiments, 500 µL of sample was aliquoted from the flasks at 0, 1, 2, 4, 6, 22, 26, 49, 74, 141, 214, 333 and 506 h after ^14^C-substrate addition and the samples placed into 1.5 mL Eppendorf tubes. The samples were centrifuged at 20 817 *g* for 3 min. A 250 µL volume of supernatant was removed and the remaining solution was discarded. A 25 µL volume of 0.1 M HCl was added to the supernatant and allowed to incubate for 3 h to remove any dissolved CO_2_ present. Finally, 4 mL of Optiphase HiSafe-3 scintillation cocktail (PerkinElmer) was added to the solution. To measure ^14^CO_2_ production, 300 µL was taken from each 1 M NaOH trap at various times over the course of the experiment and then 4 mL of Optiphase HiSafe-3 scintillation cocktail (PerkinElmer) was added to the solution. Levels of ^14^C in the solutions were measured using a Wallac 1404 liquid scintillation counter with automated quench correction (Wallac EG&G, Milton Keyes, UK). Biomass incorporation was calculated by difference using the results obtained from CO_2_ evolution and residual cell-unbound substrate concentration and assuming no ^14^C volatile losses other than ^14^CO_2_.

Mean and standard error at each time were calculated in R using the packages plyr (Wickham 2011) and sciplot (Morales *et al*. 2017). Graphs were generated in R using the ggplot2 (Wickham 2009) and gridExtra (Auguie 2015) packages. Repeated measures analysis of variance (ANOVA) was performed on the ^14^C data using SPSS Statistics 25 (IBM UK Ltd., Portsmouth, UK) to measure the effects of treatment over the 3-week period (Table S2, see online supplementary material). The Mauchly's test for sphericity was also performed. However, all values were non-significant (*P* > 0.05). The Greenhouse–Geisser estimate of sphericity was done to determine the proper correction value. If ε < 0.75, the Greenhouse–Geisser correction was applied and if ε > 0.75, the Huynh–Feldt correction was applied (Table S2). *Post hoc* multiple pairwise testing was carried out using Tukey's *post hoc* multiple pairwise testing. The Games–Howell test was applied if the assumptions of the ANOVA test were not met.

### 16S rRNA amplicon preparation, sequencing and statistical analysis

Samples of river water (500 µL) were removed from the flasks at 0, 49, 141, 333 and 506 h and subsequently centrifuged (21 000 *g*, 10 min) removing the supernatant. The remaining pellet was then washed (x3) with phosphate buffer solution (PBS) (pH 7.4). For the preparation of Illumina-compatible libraries of the V4 region of 16S rRNA gene, a dual-indexing primer system with heterogeneity spacer was used (Fadrosh *et al*. 2014). The rRNA-annealing parts of the primers corresponded to standard F515-R806 primers with slight modifications aimed to improve the coverage of environmental taxa (Table S3, see online supplementary material). All PCR reactions were performed in a Bio-Rad^®^ thermocycler with the following program: 95°C for 2 min for denaturation followed by 33 annealing cycles, 95°C for 45 s, 50°C for 1 min, 72°C for 30 s and finally 72°C for 3 min. PCR products were checked using gel electrophoresis (1.8% agarose gel). A QIAquick gel extraction kit^®^ (Qiagen) was used to purify PCR fragments from the agarose gel. A Qubit^®^ dsDNA HS kit (Life Technology) with Qubit^®^ Fluorometer was used to determine the concentration of DNA. Samples were then subsequently dried down via spin vacuum. The barcoded amplicons were sequenced with a MiSeq™ benchtop sequencer (Illumina Inc., San Diego, CA, USA) using paired-end 250 bp reads. All next generation sequencing (NGS) reads were subjected to stringent quality filtering, and parts of reads corresponding to 16S rRNA primers were removed using CLC Genomics Workbench 10.0 (Qiagen, Germany). After quality trimming, overlapping paired reads were merged with the SeqPrep tool (https://github.com/jstjohn/SeqPrep). All parameters were default, except the maximum fraction of good quality mismatching bases to overlap reads was set to 0.05.

Further processing, including demultiplexing, operational taxonomic unit (OTU) generation and taxa assignment, was performed with the Qiime bioinformatics pipeline (Caporaso *et al*. 2010). Generation of OTUs was performed with the open-reference algorithm (script pick_open_reference_otus.py). OTU processing is described further here https://github.com/RafaBargiela/MiSeqDualIndx. Taxa assignment was performed using 97% identity clustered sequences of the Silva128 database (Yilmaz *et al*. 2014).

The R programming language (R Core Team 2017) was used for statistical analysis and figure creation (Wickham 2009). Non-metric multidimensional scaling (NMDS), using the Bray–Curtis calculation method, was used to examine beta diversity via the phyloseq package (McMurdie and Holmes 2013). Rarefaction curves were created using the ranacapa package (Kandlikar *et al*. 2018). Ap ermutation multivariate analysis of variance (PERMANOVA) via a mixed effect model was calculated from the relative abundances using the Bray–Curtis method with 999 permutations via the vegan package (Oksanen *et al*. 2018). Then subsequent stepwise model selection was utilized to determine which effects/mixed effects had the greatest influence on OTU absence/presence.

### Targeted metabolomic analysis by a gas chromatography electron ionization quadrupole time-of-flight mass spectrometry system

Aliquots (10 mL) from the substrate depletion samples (see above) were recovered from the flasks at 0, 49, 141, 333 and 506 h after substrate treatment and stored at −86°C. The concentration per each substrate per sample (microcosm) is listed in Table S1. The samples were freeze-dried and re-suspended in 0.2 mL of dH_2_O then stored at −86°C until use. Aliquots (70 µL) were evaporated to dryness using a SpeedVac^®^ Concentrator and treated with 10 μL of O-methoxyamine hydrochloride (15 mg/mL) in pyridine. Subsequently, the vials were incubated in darkness at room temperature for 16 h, 10 μL of N, O-bis(trimethylsilyl)trifluoroacetamide (BSTFA) with 1% (v/v) trimethylchlorosilane (TMCS) was added and vortexed for 5 min. Silylation was carried out for 1 h at 70°C and samples were treated with 100 μL of C18:0 methyl ester (10 mg/L in heptane). The gas chromatography (GC) system (Agilent Technologies 7890B) consisted of an autosampler (Agilent Technologies 7693) connected to an accurate-mass quadrupole time-of-flight (Q-TOF) mass spectrometer (Agilent Technologies). A 2 μL volume of the derivatized sample was injected onto a DB5-MS column (30 m length, 0.25 mm internal diameter, 0.25 μm film 95% dimethylpolysiloxane/5% diphenylpolysiloxane) with a pre-column (10 m J&W integrated with Agilent 122–5532G). The flow rate of the He carrier gas was set at 0.85 mL min^−1^ and the injector temperature 250°C. The split ratio was 1:12. The temperature gradient was programmed at 60°C (held for 1 min), with a ramping increase rate of 10°C/min up to 325°C. Finally, the instrument was cooled down for 10 min before the next injection. The total analysis time was 37.5 min. The electron ionization (EI) source was placed at 70 eV. The mass spectrometer operated in scan mode over a mass range of m/z 50–600. The method was retention-time locked at 19.66 min (elution time of the internal standard). The analytical run was set up starting with the injection of C18:0 methyl ester (10 mg/L in heptane) followed by three blanks, and then samples were analysed in a randomized order, until the end of the run that terminated with the injection of the three blanks. The relative abundance based on peak intensity was used for downstream applications (Table S4, see online supplementary material). The absolute value of the rate of change between several time intervals was calculated (Table S5, see online supplementary material). Homoscedastic Student's t-testing with two-tailed distribution was done on the initial and final time points to check for depletion of added substrates (Table S6, see online supplementary material).

### Shotgun sequencing

Samples of river water (500 µL) were removed from the flasks at 0, 141 and 506 h and centrifuged (21 000 *g*, 10 min), removing the supernatant. Whole genome amplification (WGA) was achieved using the REPLI-g UltraFast Mini kit (Qiagen, Germany) as per the manufacturer's protocol. DNA (∼1 µg) was sheared using a Bioruptor Pico sonicator (Diagenode) by sonicating at 4°C with four cycles of 15 s on and 90 s off, to obtain fragments of 600–800 bp size. Library preparation was performed using the NEBNext^®^ Ultra™ II DNA Library Prep Kit (New England Biolabs) according to the protocol provided by the manufacturer. Briefly, fragmented DNA was end-repaired and ligated to the Illumina adaptor. Adaptor-ligated DNA was amplified with index primers provided in NEBNext Multiplex Oligos Set 1 and Set 2 (New England Biolabs). Size-selection and PCR clean-up were performed by gel purification using the QIAEX II Gel extraction kit (Qiagen, Germany). Subsequently, the barcoded libraries were quantified using a Qubit^®^ dsDNA HS Assay Kit with a Qubit 4 Fluorometer. Samples were then pooled in equimolar amounts and the resulting pool was diluted to a final concentration of 4 nM. The final pool was denatured and sequenced using the MiSeq Reagent Kit v3, 600 Cycles Sequencing kit on the MiSeq System (Illumina). Quality control was done using *fastqc* (Andrews 2010) and adapter trimming using *cutadapt* (Martin 2011), discarding those reads with average quality <20 or shorter than 20 bps. Assembly was performed using MEGAHIT (Li *et al*. 2015). Gene prediction and annotation was done using PROKKA (Seemann 2014). Additional annotation was added using emapper and DIAMOND (Buchfink, Xie and Huson 2015) with the eggNOG database (Huerta-Cepas *et al*. 2016). Binning sample reads and assembled contigs were done using MaxBin (Wu *et al*. 2014). The resulting bins have been further classified with Kraken2 based on the NCBI RefSeq database to get taxonomic classifications (Wood, Lu and Langmead 2019). The bioinformatic pipeline is described here https://github.com/RafaBargiela/MetagenomeProcessing. Homoscedastic Student's t-testing with two-tailed distribution was performed on the data (Table S7, see online supplementary material).

### Availability of data

The shotgun sequencing dataset and relevant metadata reported in this study have been deposited in NCBI genebank Archive under Bioproject PRJNA599939 with the accession numbers SRR10850302–SRR1085032. The 16S profiling dataset and relevant metadata were submitted in NCBI SRA archive under Bioproject PRJNA625512 as merged V4 amplicon reads with accession numbers SRR11549683–SRR11549732.

## RESULTS

### Metabolic activity measured by ^14^C-DOC depletion and targeted metabolomics

**Figure 1.**
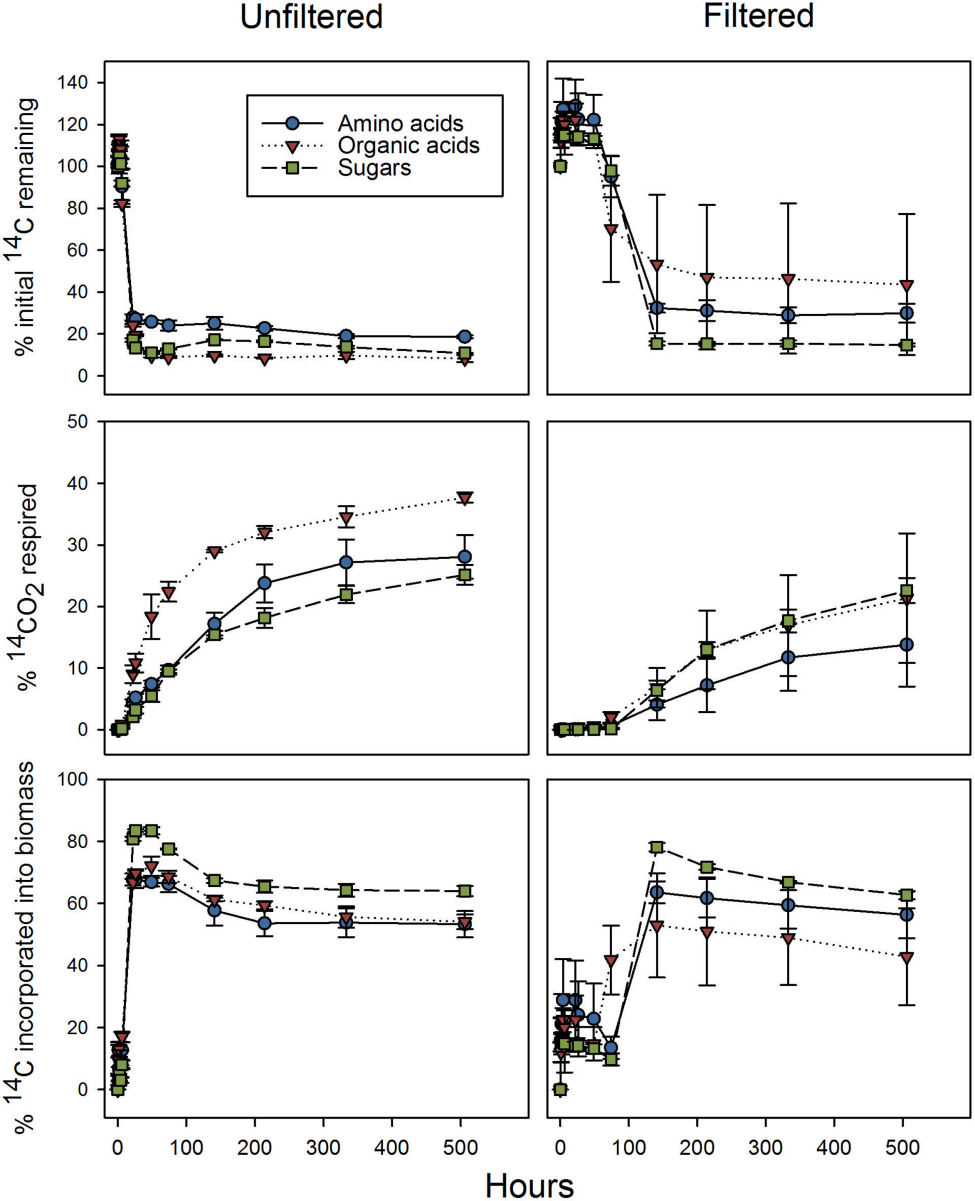
^14^C-Labelled substrate utilisation in 0.22 µm filtered and unfiltered Conwy River water. The panels show: upper, substrate depletion; middle, cumulative ^14^CO_2_ production (respiration); and lower, amount of ^14^C immobilized in the microbial biomass. Values represent means ± standard error (*n* = 3).

Overall, average rates of consumption for each of the three ^14^C-labelled substrate groups was similar (Fig. 1 and Table S5). However, there were significant differences observed between the two fractions. Firstly, the unfiltered microbiome started consumption and incorporation of carbon into the biomass within 22 h of starting the experiment, that then plateaued, whereas the filtered fraction had a delay in uptake and respiration until 74 h, which then plateaued after 141 h (Fig. 1 and Table S5). Within the filtered fraction, there was a clear lag phase across all substrates in the first 74 h (Fig. 1). Then from 74 to 141 h we observed a spike in metabolic activity across all substrate types until it slows at 214 h. Repeated measures ANOVA showed a significant difference (*P* ≤ 0.001) between treatments and measurement time (Table 1). According to the *F*-values, there was a much larger effect due to treatment alone than the compounded effect of treatment and incubation time (Table 1).

**Table 1.** Summary of the repeated measures ANOVA. Results used to determine the effects of filtering treatment and time on the use of three different ^14^C-labelled substrates by Conwy River water microcosms. *F*-values, *P*-values and degrees of freedom (df) are reported based on correction from the Maulchy's test of sphericity (Table S2).

Measurement	Substrate	Effect	Correction applied	df	*F-value*	*P-*value
^14^C Substrate depletion	^14^C Amino acids	Treatment	Greenhouse–Geisser	1.444	267.258	<0.001
^14^C Substrate depletion	^14^C Amino acids	Treatment x duration	Greenhouse–Geisser	17.322	13.085	<0.001
^14^C Substrate depletion	^14^C Organic acids	Treatment	Greenhouse–Geisser	1.177	73.700	<0.001
^14^C Substrate depletion	^14^C Organic acids	Treatment x duration	Greenhouse–Geisser	14.126	4.381	0.001
^14^C Substrate depletion	^14^C Sugars	Treatment	Greenhouse-Geisser	1.074	217.896	<0.001
^14^C Substrate depletion	^14^C Sugars	Treatment x duration	Greenhouse–Geisser	12.884	19.531	<0.001
^14^CO_2_ production	^14^C Amino acids	Treatment	Huynh–Feldt	2.000	143.215	<0.001
^14^CO_2_ production	^14^C Amino acids	Treatment x duration	Huynh–Feldt	24.000	13.701	<0.001
^14^CO_2_ production	^14^C Organic acids	Treatment	Greenhouse–Geisser	1.125	105.501	<0.001
^14^CO_2_ production	^14^C Organic acids	Treatment x duration	Greenhouse–Geisser	13.497	7.175	<0.001
^14^CO_2_ production	^14^C Sugars	Treatment	Greenhouse–Geisser	1.351	326.369	<0.001
^14^CO_2_ production	^14^C Sugars	Treatment x duration	Greenhouse–Geisser	16.210	43.863	<0.001
^14^C Biomass incorporation	^14^C Amino acids	Treatment	Greenhouse–Geisser	1.840	12.317	<0.001
^14^C Biomass incorporation	^14^C Amino acids	Treatment x duration	Greenhouse–Geisser	22.084	6.429	<0.001
^14^C Biomass incorporation	^14^C Organic acids	Treatment	Huynh–Feldt	2.000	82.803	<0.001
^14^C Biomass incorporation	^14^C Organic acids	Treatment x duration	Huynh–Feldt	24.000	6.126	<0.001
^14^C Biomass incorporation	^14^C Sugars	Treatment	Huynh–Feldt	1.604	140.036	<0.001
^14^C Biomass incorporation	^14^C Sugars	Treatment x duration	Huynh–Feldt	19.242	20.388	<0.001

As for each substrate type, there is not a clear discernible preference across all substrate types due to the level of variability seen in the filtered fraction (Fig. 1). Significant interactions (*P* ≤ 0.001) between the samples (whole community versus filtered) and experiment duration were observed for all ^14^C-labelled substrate types (i.e. amino acids, organic acids and sugars) (Fig. 1 and Table S2). Our blanks (negative controls) showed no signs of metabolic activity in comparison to the other treatments.

**Figure 2.**
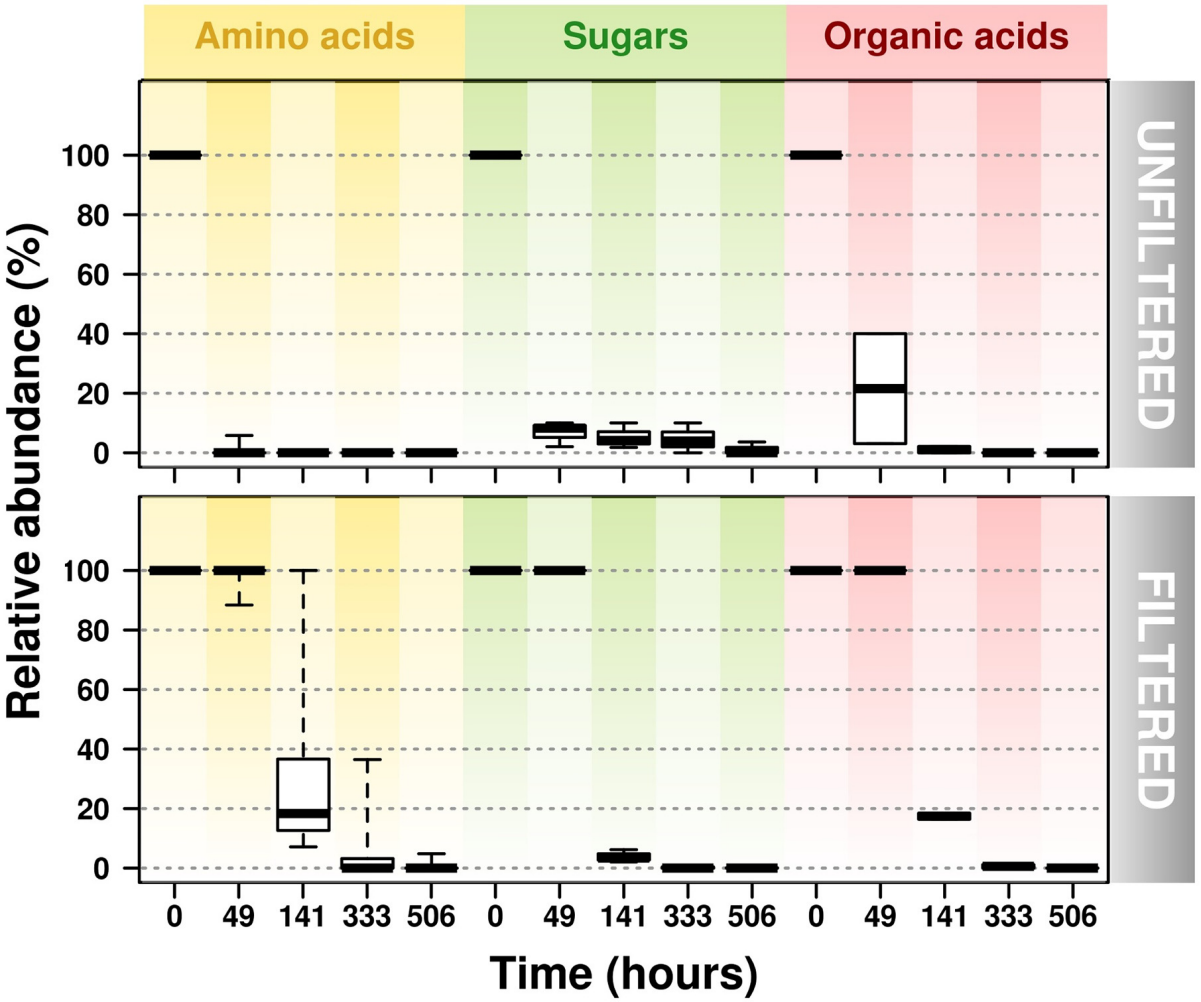
Residual concentrations of LMW substrates added to microcosms in filtered and unfiltered samples upon incubation for 506 h. A total of 16 LMW substrates (see Methods for details), 11 amino acids, 3 carbohydrates and 2 organic acids, were quantified by GC-MS in biological triplicates.

Targeted metabolomics quantified 16 metabolites from the three substrate types spiked to the river water at nanomolar concentrations. We used a GC-EI-QTOF-mass spectrometry (MS) system that has a detection limit much below this concentration (ppm or mg/L). This included the amino acids: alanine, aspartic acids, glycine, isoleucine, leucine, phenylalanine, proline, serine, threonine, tyrosine and valine; the organic acids: citric acid and malic acid; and the sugars: fructose, glucose and sucrose. Overall, these showed very similar depletion patterns to those measured using the ^14^C-labelled substrates (Figs 2 and S1, see online supplementary material, and Table S6). In contrast to the ^14^C results, however, no detectable substrates remained in solution of both microcosms at the end of the experiment (Figs 2 and S1).

### Microbial community composition (16S rRNA amplicon sequencing)

Bacteria and archaea communities at various timepoints (0, 49, 141, 333 and 506 h) in experimental and unamended samples were examined using analysis of 16S rRNA V4 amplicon sequencing. Sequencing libraries were successfully prepared and sequenced for 51 of the 61 samples (Table S8, see online supplementary material). In total, there were 668 405 reads across all the samples and time points (306 244 in the filtered fraction, 362 161 in the unfiltered sample) (Table S8). Resulting data were analysed by a classical OTU-based approach using the Silva128 16S rRNA database for taxonomy assignment (See Methods).

**Figure 3.**
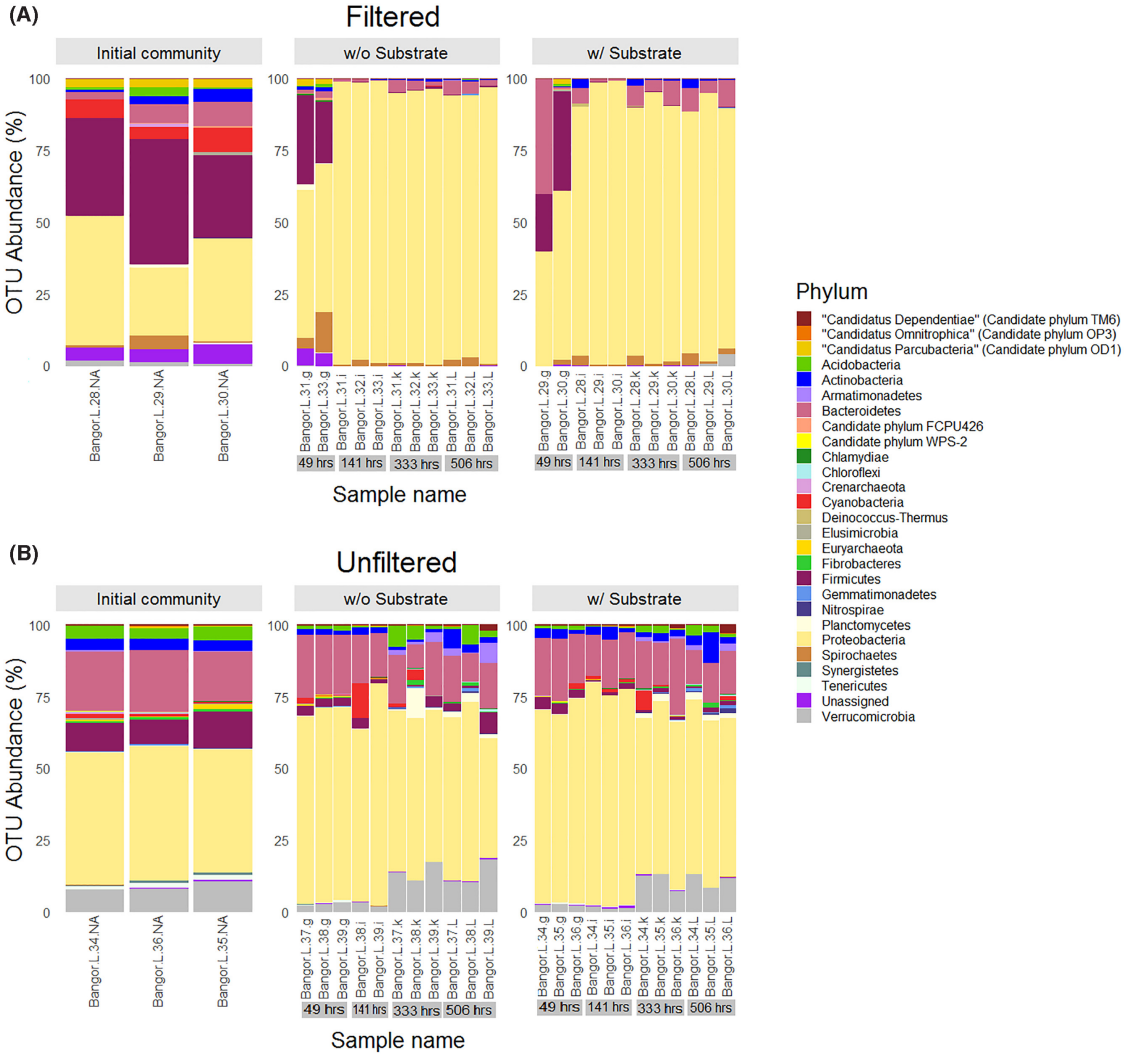
Phyla distribution with high-abundance OTUs between treatments and substrate additions in mesocosms upon incubation for 506 h. (**A**) Filtered fraction and (**B**) total (unfiltered) community. Left panel shows composition in the initial communities (*n* = 3). Middle panels show the communities changing over the course of the 3 weeks without (w/o) additional substrate versus the right column with (w/) additional substrates. Refer to Table S7, Fig. S1 and Fig. S2 for further information regarding lower order taxa. Abundances lower than 50 gene counts were removed. See Methods section for more detail.

Large differences in taxonomic composition and abundance of OTUs were apparent between the filtering treatments and measurement times (Fig. 3). Phyla that were prominent in the filtered fraction of the initial communities were *Proteobacteria, Firmicutes, Spirochetes, Actinobacteria, Bacteroidetes, Acidobacteria*, ‘*Candidatus* Parcubacteria’ (Candidate phylum OD1) and unassigned groups. As the experiment progressed, *Proteobacteria* became the dominant phylum as *Firmicutes*, ‘*Ca*. Parcubacteria’, *Spirochetes, Cyanobacteria, Acidobacteria, Actinobacteria* and unassigned groups decreased. In comparison, the unfiltered community was composed mainly of *Proteobacteria, Bacteroidetes, Actinobacteria, Armatimonadetes, Verrucomicrobia, Acidobacteria* and *Firmicutes* in the initial community (Fig. 3). *Firmicutes* decreased over the course of the 3-week experiment while the proportion of *Actinobacteria, Armatimonadetes* and *Verrucomicrobia* increased. The appearance of minority phyla such as ‘*Ca*. Dependentiae’ (Candidate phylum TM6) and ‘*Ca*. Omintrophica’ (Candidate phylum OP3) were dependent on whether substrate was added to the sample (Fig. 3). The only archaeal group detected in very small quantities was *Euryarchaeota* (Fig. 3).

Analysis of OTUs taxonomic distribution on the class and family taxonomic levels showed that at the initial stages of the experiment, the filtered fraction was dominated by *Bacilli, Betaproteobacteria, Gammaproteobacteria, Deltaproteobacteria, Spirochaetes, Alphaproteobacteria, ZB2, Actinobacteriia* and *Cyanobacteria* (Fig. S2 and Table S9, see online supplementary material). Upon further inspection at the family level, *Bacillaceae* (*Bacilli, Bacillales*), *Halomonadaceae* (*Oceanospirillales, Gammaproteobacteria*) and unassigned groups (i.e. unidentified taxa) were dominant (Fig. S2 and Table S9). On the other hand, the initial unfiltered community was predominantly composed of *Betaproteobacteria, Alphaproteobacteria, Bacteroidia, Clostridia, Flavobacteriia, Gammaproteobacteria, Sphingobacteriia* and *Bacilli*; with a fairly even distribution across a plethora of families, with the most dominant being *Oxalobacteraceae* (*Burkolderiales, Betaproteobacteria*), *Comamonadaceae* (*Burkolderiales, Betaproteobacteria*), *Flavobacteriaceae* (*Flavobacteriales, Flavobacteriia*), *Verrucomicrobiaceae* (*Verrucomicrobiales, Verrucomicrobiae*) and unassigned families (Figs S2, S3 and Table S9, see online supplementary material). During the later stages of the experiment (49–506 h), the filtered fraction was almost completely dominated by *Betaproteobacteria, Gammaproteobacteria* and *Epsilonproteobacteria* (Fig. S2 and Table S9). More specifically, the most prevalent families were *Comamonadaceae, Campylobacteraceae* (*Campylobacterales, Epsilonproteobacteria*), *Oxalobacteraceae* and *Pseudomonadaceae* (*Pseudomonadales, Gammaproteobacteria*) (Fig. S3 and Table S9). However, the microbial community in the unfiltered fraction remained relatively consistent in terms of distribution at both class and family levels, with the exception of *Bacteroidia*, which were only found in the first 49 h, and *Verrucomicrobiaceae*, which increased in the latter stages of the experiment (Fig. S3).

**Figure 4.**
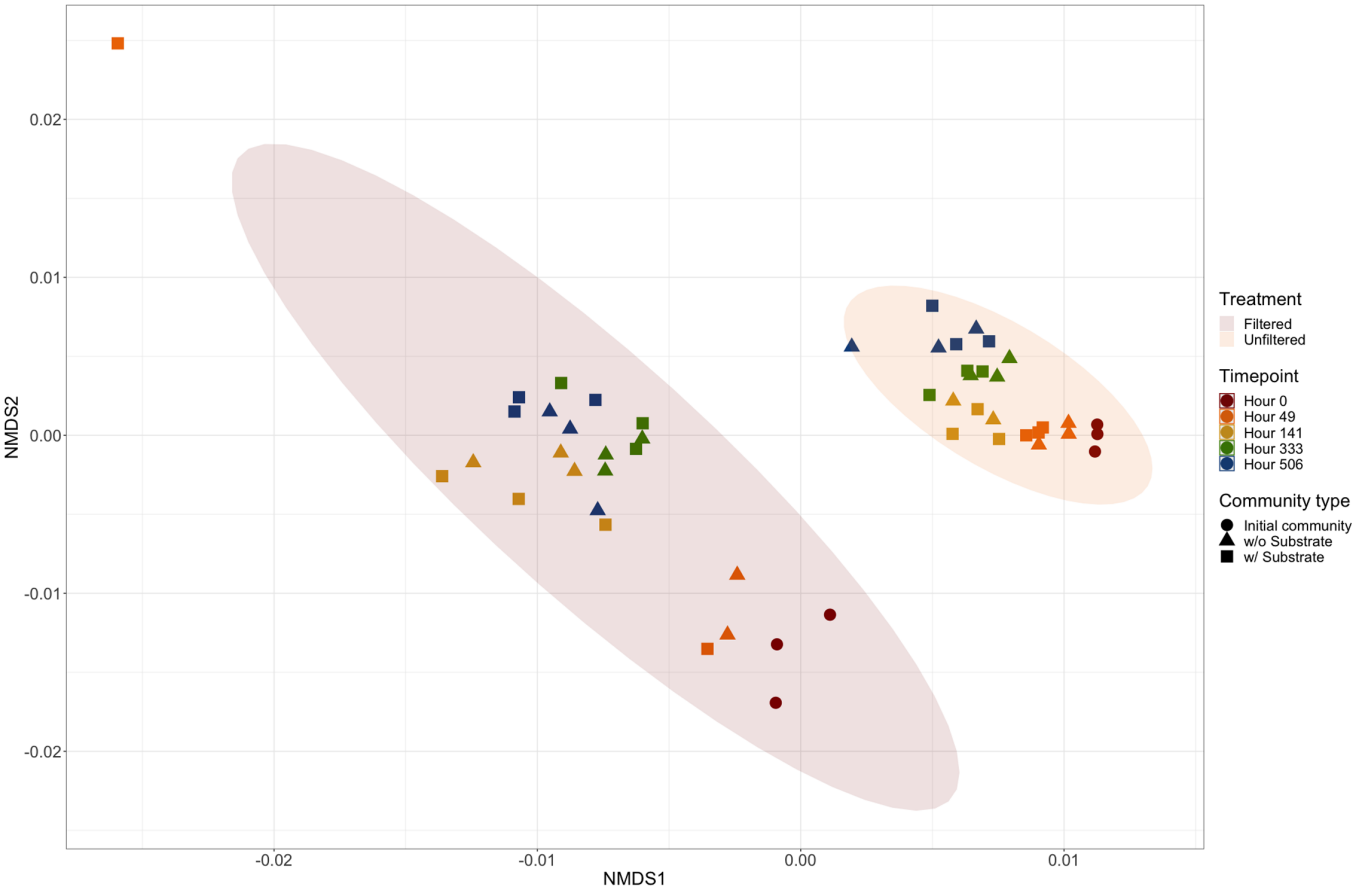
Microbial beta diversity in all samples and time points (49, 141, 333 and 506 h). NMDS plotted using the Bray–Curtis calculation method (stress value = 0.094). w/o, without; w/, with. See Methods section for more details.

**Figure 5.**
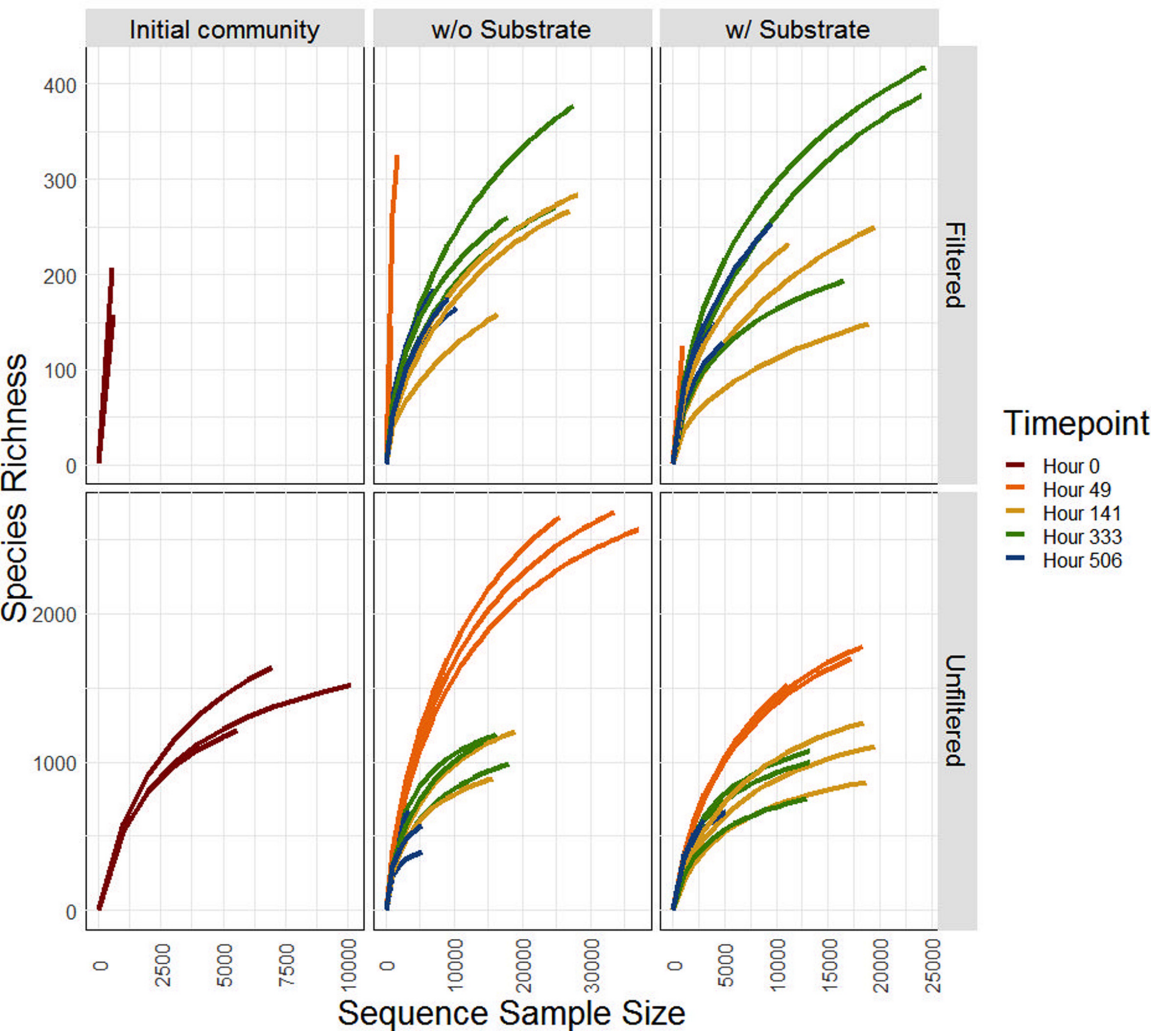
Rarefaction curves for samples and time points (49, 141, 333 and 506 h). The top panel is the filtered community and the bottom is the total community (unfiltered fraction). The left column shows the initial community, the middle is the community from 49–506 h without substrate addition, and the right-hand column is the community from 49–506 h with substrate addition.

A PERMANOVA test was used to compare the effects of substrate addition, duration and treatment on the relative abundance of OTUs present in the samples. Overall, the addition of substrate had no measurable effect on OTU abundance (*P* > 0.05), whereas treatment and duration had significant effects on the OTUs present (*P* < 0.05). After subsequent stepwise model selection, the greatest effect on the presence and absence of OTUs was a mixed effect of both treatment and experiment duration (*P* < 0.05). Examining alpha (rarefaction) and beta diversity by NMDS showed that there were measurable differences between both fractions and experimental time points (Fig. 4). There was no noticeable effect of low-molecular-weight DOC addition on community composition (Fig. 4). Based on the rarefaction curves (Fig. 5), there seems to be more diversity in the unfiltered community versus the filtered. The initial communities and those measured at 49 h in both river water fractions were more diverse than those measured towards the end of the experiment (141–506 h) (Fig. 5). We note that within both fractions, diversity decreases over the 3-week experimental period (Fig. 5).

In relation to taxa that have been linked to being potential ultramicrobacteria, at the phylum level ‘*Ca*. Parcubacteria’, ‘*Ca*. Ominitrophica’ and ‘*Ca*. Dependentiae’ were the major groups (Fig. 3). Of those three Ca. Parcubacteria was the most prevalent, especially within the filtered samples, followed by ‘*Ca*. Dependentiae’ and ‘*Ca*. Ominitrophica’. Interestingly, no other commonly associated groups such as freshwater SAR11 (also referred to as LD12 subclade) and ac1 *Actinobacteria* were detected in the raw or filtered samples.

### Assessment of clusters of orthologous groups categories of both fractions and taxa assignments (shotgun DNA sequencing data)

The entire communities prior to and after substrate addition were examined using shotgun DNA sequencing. Three time points (0, 141 and 506 h) were chosen to examine the changes of the community metagenome. The number of contigs was variable, ranging from 25,762 to 256,700 across all treatments, time points and substrate amendments; with the whole community containing overall more contigs than the filtered community (Table S9). Here, we compared across microcosm samples the numbers of genes encoding proteins that fall under the functional categories of clusters of orthologous groups (COGs). The number of COG assignments across all sample types ranged from 893 to 99,402. The overall abundance of COGs within the whole community were not dependent on substrate addition nor experiment duration (Table S7). However,t -tests revealed that adding substrate had a greater effect on the distribution of functional categories within the filtered fraction (*P* < 0.05) (Table S7).

**Figure 6.**
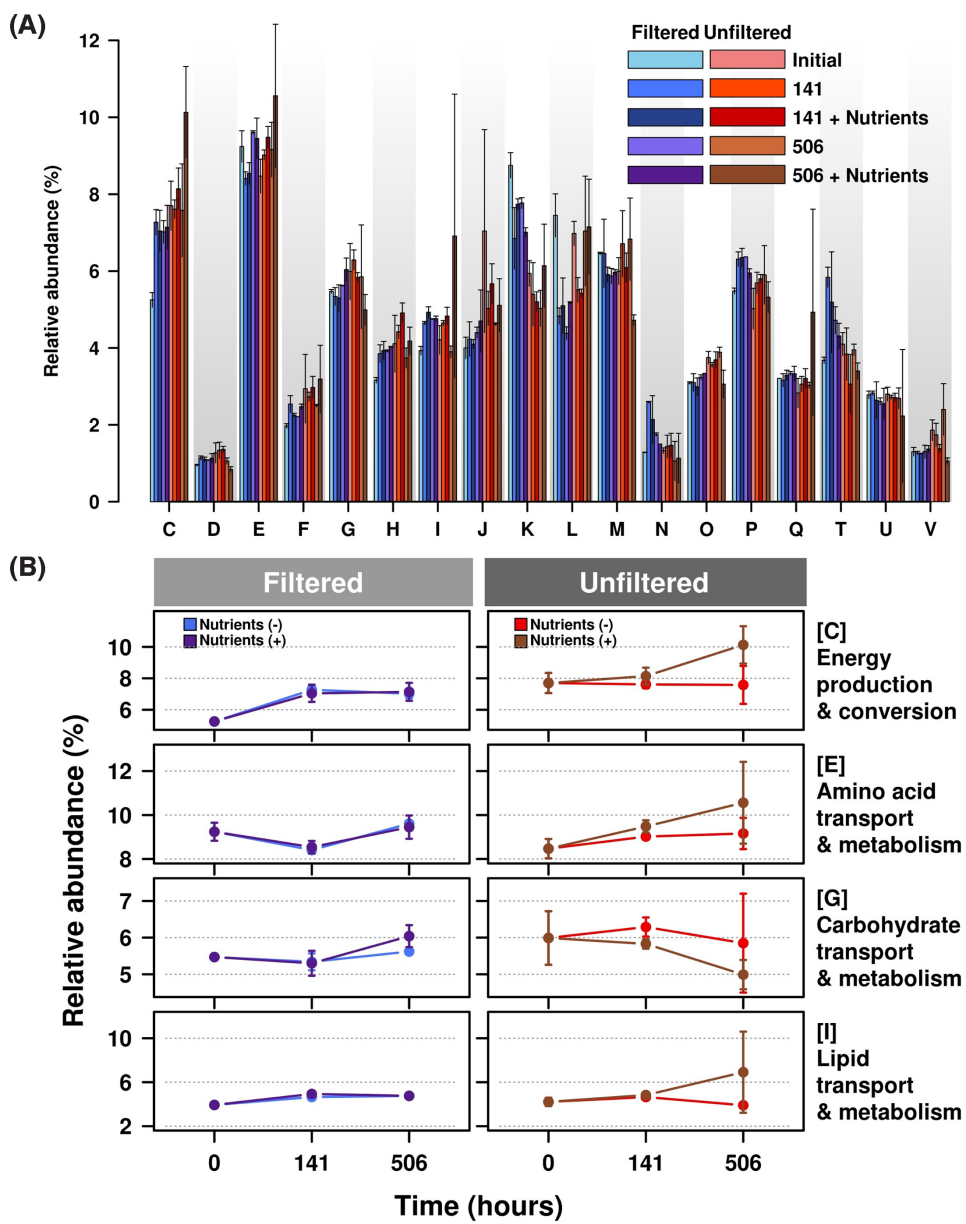
Distribution of functional categories of COGs derived from shotgun DNA sequencing. (**A**) The relative abundance (%) of total gene count that falls under a COG category is compared between the filtered (cool/blue colours) and unfiltered (warm/red colours) fraction over the course of 506 h with and without substrate amendments. The letter designations in the horizontal axis are as follows: J, translation, ribosomal structure and biogenesis; K, transcription; L, replication, recombination and repair; D, cell cycle control, cell division, chromosome partitioning; V, defense mechanisms; T, signal transduction mechanisms; M, cell wall/membrane/envelope biogenesis; N, cell motility; W, extracellular structures; U, intracellular trafficking, secretion, and vesicular transport; O, posttranslational modification, protein turnover, chaperones; C, energy production and conversion; G, carbohydrate transport and metabolism; E, amino acid transport and metabolism; F, nucleotide transport and metabolism; H, coenzyme transport and metabolism; I, lipid transport and metabolism; P, inorganic ion transport and metabolism; and Q, secondary metabolites biosynthesis, transport and catabolism. Values represent means ± standard deviation (*n* = 2). (**B**) COGs associated with DOM usage and energy production. Energy production and conversion, amino acid transport and metabolism, carbohydrate transport and metabolism, and lipid transport and metabolism were selected. The relative abundance (%) of total gene count that falls under a COG category is compared between the filtered (warm colours) and unfiltered (cool colours) fraction over the course of 506 h with and without substrate amendments. Values represent means ± standard deviation (*n* = 2).

Examination of the COG assignments across both fractions and nutrient amendments over the course of the 3 weeks showed some notable trends. Namely, COGs that were affiliated with specific metabolic pathways (i.e. utilization of amino acids, carbohydrates and lipids) did not vary in either fraction over the course of the 3-week period regardless of substrate amendment (Fig. 6). Rather, the percentage of general COGs affiliated with energy production and conversion increased in both fractions with substrate addition (Fig. 6). The other COGs in the filtered fraction were influenced by the presence of added substrate such as those affiliated with amino acid metabolism; coenzyme transport and metabolism; translation, ribosomal structure and biogenesis; cell motility; inorganic ion transport; secondary metabolism; signal transduction; post-translational modification, protein turnover and protein chaperones; and unknown functionalities (Fig. 6 and Table S7). On the other hand, the COGs present in the whole community were not influenced by the presence of nutrient amendments (Fig. 6 and Table S7).

**Figure 7.**
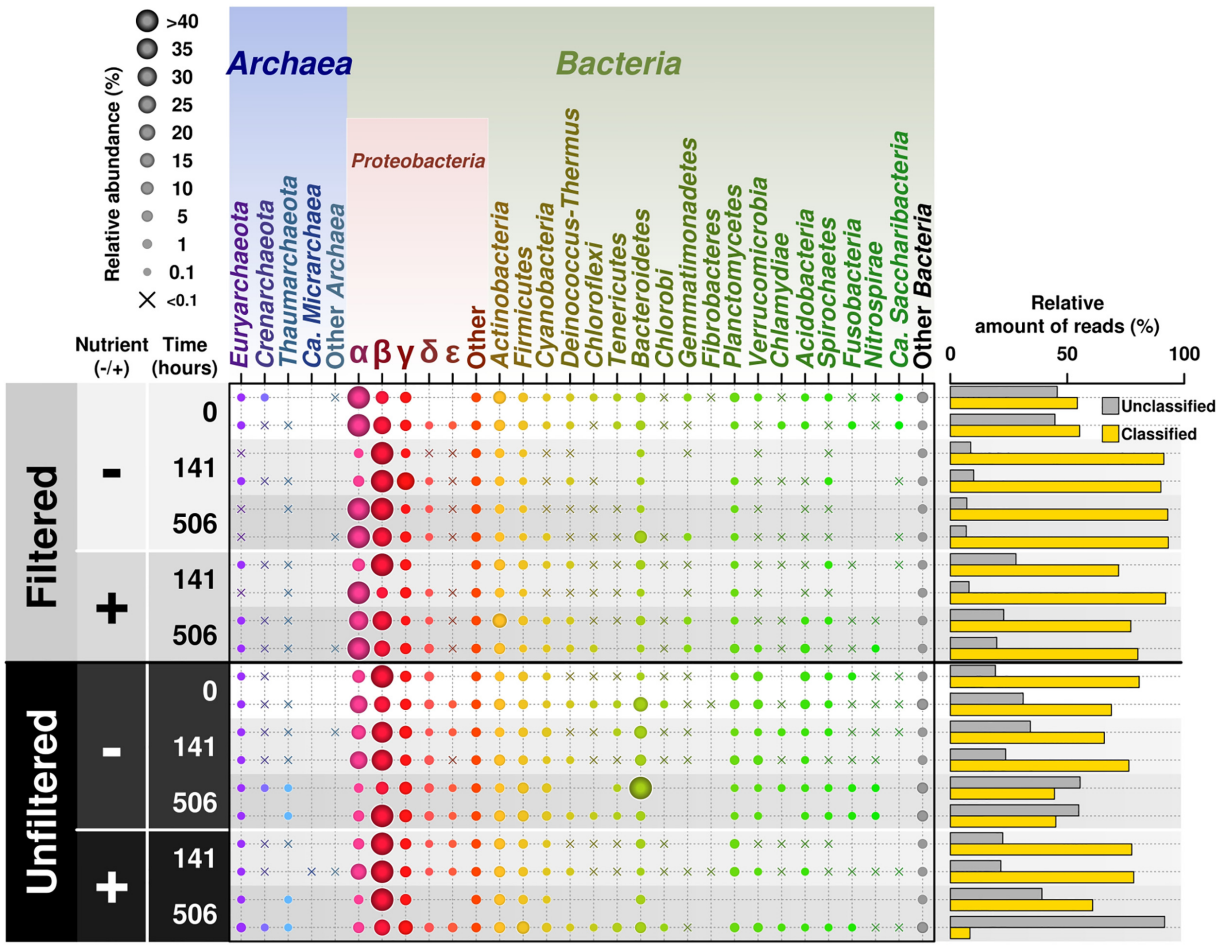
Relative abundance of phyla detected via shotgun sequencing comparing treatments and substrate additions over 506 h. The top and bottom panels show data for filtered fraction and total community (unfiltered fraction). Samples (*n* = 2) dependent on nutrient amendment (−/+ nutrients) and time point (0 h, 141 h and 506 h) were measured. Percentages of classified versus unclassified reads in each sample are shown on the right.

As for taxonomic assignments, there was a greater percentage of unclassified reads in the total community versus the filtered fraction, especially at 506 h (Fig. 7). The most prominent groups closely mirrored those of the 16S rRNA metabarcoding data, where *Proteobacteria* was the most dominant phylum in both fractions, specifically *Alphaproteobacteria, Betaproteobacteria* and *Gammaproteobacteria* (Fig. 7). *Betaproteobacteria* dominated in the entire community, followed by *Alphaproteobacteria* and *Gammaproteobacteria*. Similarly to the 16S rRNA barcoding, *Actinobacteria* and *Bacteroidetes* were more prevalent in the total community. Interestingly, *Firmicutes*, which were dominant in the filtered fraction of the initial phases according to the 16S rRNA metabarcoding, showed similar relative abundances to *Actinobacteria* in both filtered fraction and entire community. *Euryarchaeota* was the dominant archaeal phylum within both sample types, especially at the beginning of the experiment. Then *Crenarchaeota* and *Thaumarchaeota* increased in abundance within all the samples over the course of 3 weeks where these were in equal prevalence to *Euryarchaeota* (Fig. 7). Other *Archaea* (unidentified groups) and ‘*Ca*. Micrarchaea’ were found in minor numbers at the end phase of the experiment (Fig. 7).

## DISCUSSION

### Utilization of DOC and fractions of the Conwy River

LMW compounds (amino acids, organic acids and sugars) were chosen for this study because (i) they represent major constituents of DOM and (ii) they are a ubiquitous source of nutrients for many heterotrophic freshwater species. The river water in previous studies prior to addition of substrates showed a significant amount of DOC present (Emmett *et al*. 2016; Brailsford et al. 2017, 2019). However, it is hard to determine the form C is in because the definition of DOM also includes particulates that have the ability to pass through ultra-small filters (filter sizes <0.45 µm) (Brailsford *et al*. 2017). Therefore, specific substrates were used to determine C utilization by the intrinsic microbial community.

Both the filterable fraction and the whole community fraction possessed the ability to use LMW compounds (Figs 1, 2 and S1). The majority of the substrate was utilised within 2 days in the unfiltered freshwater, however, in the filtered fraction substrate depletion took much longer. This lag phase in the filtered samples occurred independently of the type of substrate added (Figs 1, 2 and S1). Similar lag-phase responses have also been observed in marine systems (e.g. the Mediterranean and Baltic Seas), where bacterial growth occurred within 1–3 days after micromolar additions of LMW DOC (Gómez-Consarnau *et al*. 2012) and also in deep subsoils from the Conwy catchment (de Sosa *et al*. 2018). Marine systems (especially pelagic areas) and major stretches of the Conwy River are classified as oligotrophic (Alonso-Sáez and Gasol 2007; Gómez-Consarnau *et al*. 2012). Organisms adapted to oligotrophic environments decrease in size to maximize surface area for nutrient consumption. However, when nutrients are introduced into the system, copiotrophs (i.e. microorganisms that prefer high nutrient, eutrophic environments) over proliferate and cells may increase in size (Ghuneim *et al*. 2018). Therefore, the lag phase observed is probably the result of two factors. Firstly, there was a very low abundance of microorganisms in the filtered fraction, due to removal of larger microorganisms. Consequently, the lag phase might simply reflect microbial re-growth (Jiang, Ni and Zhang 2011). Secondly, the microorganisms passing through the filter may have been metabolically inactive, which is typical for starved cells. The time for bacterial reactivation seen in other water systems is consistent with the duration of the lag-phase response observed in our previous study (Lin *et al*. 2016). The duration of the lag phase is important in ecological terms considering the residence time of the water in the Conwy catchment. Models suggest that water from the headwater streams reaches the coast within 1–3 days depending upon rainfall intensity (Robins *et al*. 2018). We conclude that in short catchments like the Conwy, the filtered fractions play a very minor role in DOC transformation.

Neither the unfiltered community nor its filterable component showed a specific preference for substrate type, suggesting that microorganisms in the Conwy River are generalists with regard to LMW DOC. This is evident as there was no discernible difference in rate of consumption nor the COG assignments from shotgun sequences (Figs 1 and 5). In terms of COG assignments, both microcosm types have a similar distribution of COGs related to LMW DOC cycling (Fig. 6). The increased percentage of COGs associated with functionalities related to energy production/conversion after substrate addition further supports this generalist view (Fig. 6).

### Contribution to LMW DOC utilization of various taxa

*Firmicutes*, according to the 16S rRNA barcoding, was one of the most abundant phyla in the initial phases of the experiment, especially in the filtered fraction (Figs 3, S2 and S3). It could be that the *Firmicutes* represented here are endospores (i.e. dormant forms) rather than metabolically active cells. Endospores are usually smaller, or the same size as, the original bacteria, as in the case of *Bacillus subtilis* where the bacterial cell itself is 4–10 µm long and 0.25–1.0 µm in diameter but its endospore form is 0.89–1.53 µm long and 0.41–0.67 µm in diameter (Carrera *et al*. 2007; Yu *et al*. 2014). This fact, in conjunction with ^14^C-labelled DOC measurements and the targeted metabolomics showing limited activity in the first 74 h, is strong evidence to support this (i.e. a lag phase while spores germinate and become active). Another notable factor contributing to the noted decline in population observed in all samples may stem from their anaerobic lifestyle. The constant exposure to oxygen, like that in the experiment, would be detrimental to their survival.

*Spirochetes* followed the same pattern as the *Firmicutes* in the filtered fraction. In the initial phase of the experiment, these bacteria were initially present but declined over time (Figs 3, S2 and S3). *Spirochetes*, due to its morphology (3–500 µm long and 0.09–3 µm in diameter), can easily squeeze through 0.22 µm filters relatively unharmed (Hahn 2004; Wang et al. 2007, 2008). Similar to the *Firmicutes*, the constant exposure to oxygen at regular intervals may be the reason for the noted decline in *Spirochetes*. This suggests that both these phyla present in the initial phases may not be metabolically active due to the sub-optimal growth conditions for these taxa.

*Bacteroidetes* and *Actinobacteria* were found across all samples, but they were much more prevalent in the unfiltered (whole) community (Figs 3, 7, S2 and S3). However, their numbers in the filtered fraction declined sharply over the 3-week incubation. It should be noted that in aquatic *Actinobacteria*, specifically ac1, cell volumes can be <0.1 µm^3^ (Jooste and Hugo 1999; Pernthaler *et al*. 2001; Thomas *et al*. 2011; Ghai *et al*. 2013; Salcher, Posch and Pernthaler 2013). Although similar freshwater systems (oligotrophic prealpine lakes, etc.) have ac1 as a dominant taxa (Salcher, Posch and Pernthaler 2013), the Conwy River was dominated by *Microbacteriaceae, Micrococcaceae, Corynebacteriaceae* and ACK-M1. In addition, a prominent family in the Conwy River water was *Flavobacteriaceae*, which is the largest family in the phylum *Bacteroidetes* with at least 90 genera (McBride 2014). A notable quality to this family is the ability to utilize polysaccharides on the cell surface, i.e. they can bind polysaccharides and transport oligomers via the outer membrane (McBride 2014). The morphology of individual species within this family, however, is highly variable. Usually, members are rod shaped with dimensions ranging from 0.3 to 0.6 µm in diameter and 1–10 µm long, and as they age may become spherical (Jooste and Hugo 1999). Some, under specific growth conditions, become filamentous and flexible (Jooste and Hugo 1999). These dominant families of the *Bacteroidetes* found are common in freshwater systems, which also have a notable sporulation phase and or senescence (Jooste and Hugo 1999; Hahn 2004; McBride 2014; Lewin *et al*. 2016; Chopyk *et al*. 2018). The lag phase suggests that the majority of these may either (or both) are senescent or metabolically inactive.

*Acidobacteria* and *Cyanobacteria* were also notable members in the initial community of the filtered fraction and greatly decreased within that same fraction throughout the experiment (Fig. 3). *Cyanobacteria* population decline may be attributed to limited exposure to light. As for *Acidobacteria*, they are diverse physiologically and genomically, and are found in a number of different environments (Quaiser *et al*. 2003; Barns *et al*. 2007; Kielak *et al*. 2016). Therefore, it is rather expected to find them throughout the lotic system microbiome.

*Verrucomicrobia* and *Armatimonadetes* were notable members of the unfiltered community, especially in the end stages of the experiment (Fig. 3). This result is not entirely surprising as it has been noted that in freshwater lakes, *Verrucomicrobia* abundance ranges from 1.7 to 41.7% of all bacterial sequences (Chiang *et al*. 2018). Although it has been observed that *Verrucomicrobia* numbers increase as DOM (13 µM) is added to aquatic systems, as in the case of seawater, our experiment showed no marked difference in the communities with added substrate (Landa *et al*. 2013). As for *Armatimonadetes* (Candidate phylum OP10), it is documented that some members prefer oligotrophy (Tamaki *et al*. 2011; Lee, Dunfield and Stott 2014). However, this phylum's prevalence at the end stages of the unfiltered community may signify favourable growth conditions as much of the added substrate is depleted (Fig. 2 and S1).

*Proteobacteria*, one of the most characterized bacterial phyla, was not exclusive to the unfiltered community (Figs 3, 7, S2 and S3). We also note that not all currently known *Proteobacteria* are large cells. Most famously, ‘*Ca*. Pelagibacter ubique', one of the smallest free-living cells, falls into the phylum *Proteobacteria* (Tripp 2013). There is also a freshwater variant, subclade LD12, which may occupy a similar role to its marine counterpart as it shares many similarities, i.e. preference for small consumption of small molecules and prevalence in oligotrophic environments (Salcher, Posch and Pernthaler 2013; Henson *et al*. 2018). Even though *Proteobacteria* as a whole overtook many of the sequences, especially in the initial stages of filtered fraction after 141 h (Figs 3, S2 and S3), freshwater SAR11 itself was not present in any of the samples.

Overall, the evidence suggests that *Proteobacteria* as a whole are largely responsible for DOC utilization in the filtered fraction, not *Firmicutes* or *Spirochaetes*, as this phylum was initially present in the filtered fraction after 141 h (Figs 1 –3, 7 and S1). Other aquatic systems also suggest that *Proteobacteria* were primarily responsible for LMW DOC usage. For instance when examining seawater, obtained from the Mediterranean and Baltic Seas, *Gammaproteobacteria* thrived on allochthonous carbon sources (Gómez-Consarnau *et al*. 2012). Another study within the Mediterranean (coastal waters) suggested that *Alphaprotebacteria* were the most active in terms of glucose and amino acid uptake while *Gammaproteobacteria* preferred amino acids (Alonso-Sáez and Gasol 2007).

When we further examined the family distribution of OTUs (Fig. S3) within the *Proteobacteria* phylum we found that they varied within the fractions. *Comamonadaceae* (unfiltered and filtered fractions), *Oxalobacteraceae* (filtered and unfiltered fraction), *Pseudomonadaceae* (filtered fraction), *Campylobacteraceae* (filtered fraction) and *Halomonadaceae* (filtered fraction) were the most prevalent families. These families are ubiquitous throughout the Earth's microbiome and because of this it is difficult to assign a specific functionality (Dewhirst *et al*. 1994; Williams *et al*. 2010; Baldani *et al*. 2014; de la Haba *et al*. 2014; Willems 2014; Flynn *et al*. 2017).

The lack of the LD12 subclade in the system can suggest one of two things. Firstly, due to the streamlined genome it possesses, it is difficult to detect. Secondly, that it is not as prevalent as previously thought in such oligotrophic environments, and perhaps the other candidate phyla present ‘*Ca*. Parcubacteria’, ‘*Ca*. Ominitrophica’ and ‘*Ca*. Dependentiae’ may be filling that role. Of the three, *‘Ca*. Parcubacteria’ was the most prevalent and was almost exclusively present in the initial phases (the first 49 h) of the filtered fraction, with their population declining afterwards. Much like SAR11, it has been postulated that they have reduced genomes (<1.5 Mbp), which can be an indication of a symbiotic lifestyle. For instance, a lack of biosynthetic pathways like for the synthesis of nucleic acids, vitamins and lipids, and mechanisms for DNA repair is commonplace for many symbiont species (Brown *et al*. 2015; Nelson and Stegen 2015). Even with a streamlined genome, it is suggested that this group engage in sulfur cycling in aquatic sulfur-rich environments (Harris, Kelley and Pace 2004). A recent draft genome of ‘*Ca*. Parcubacteria’ suggests that there is the potential to metabolize organic compounds (glucose, ribose, acetate) (Castelle *et al*. 2017). Further experimentation is needed to reach this conclusion.

We must also take note of the taxa distribution of the shotgun sequencing compared with the 16S rRNA barcoding. Although there were some similarities, i.e. *Proteobacteria* being the most abundant phylum, differences were observed as well. Firstly, the little to no change in overall distribution of phyla across nutrient amendments over 506 h in the shotgun sequencing versus the clear time variation seen in the meta-barcoding analysis (Figs 3 and 7). Second, the lack of *Firmicutes* in the initial phases of the experiment in the filtered fraction of the shotgun analysis versus the clear overrepresentation in the metabarcoding data (Figs 3 and 7).

Finally, the presence of other than *Euryarchaeota* archaeal groups, i.e. *Crenarchaeota* and *Thaumarchaeota* was identified (Fig. 7). It is interesting to note that these groups increased their relative abundance as the experiment progressed. We should take note that, according to 16S rRNA metabarcoding analysis, *Thaumarchaeota* (SAGMA-X group) and *Crenarchaeaota* (*Cenarchaeaceae*) OTUs were present at ultra-low abundances (Figs 3, S2 and S3, and Table S9), whereas *Euryarchaeota* families included *Methanobacteriaceae, Methanocorpusculaceae, Methanomicrobiaceae, Methanoregulaceae, Methanosarcinaceae, Methanomassiliicoccaceae*, and archaea of the order *Thermoplasmatales* were present in very low abundances (Fig. S3 and Table S9). Yet, there are inconsistencies between the NCBI and Greengenes databases regarding, inter alia, the taxonomic placement of SAGMA-X. The SAGMA-X record in NCBI and BLAST indicates 99% identity with *Nitrosotalea*. However, these abundances are not entirely unique, as it has been previously noted that archaeal groups make up <10% of the microbial community in freshwater ecosystems, but even so they effectively utilise DOM, which could explain the noted increase as a whole within the shotgun sequencing (Wells *et al*. 2006; Bomberg *et al*. 2008; Cavicchioli 2011). These differences may largely be due to inherent qualities in the analyses themselves, mainly that this metabarcoding analysis only examines 16S rRNA genes compared with shotgun sequencing which examines all the genes present in the system.

The change in the microbial community may be the result of ‘bottle effect’, i.e. significant differences of conditions *in situ* and *in vitro*: the nutrients e.g. oxygen or nitrogen sources are quickly depleted in microcosms, which causes changes in the composition of the natural microbial community (Hammes, Vital and Egli 2010). However, there are contradictory reports when it comes to the bottle effect. One study suggested that for short-term incubations (<5 days), the bottle effect is negligible, hence microbial communities do not change (Fogg and Calvario-Martinez 1989; Hammes, Vital and Egli 2010). On the other hand, another study examined the picoplanktonic communities of oligotrophic marine water over a 24 h period and observed a shift from autotrophs to heterotrophs (Calvo-Díaz *et al*. 2011). Other accounts suggest that bottle size is a determining factor (Fogg and Calvario-Martinez 1989). The consensus is that bottle effect is something to account for, depending on the conditions (such as initial sample composition, ambient light, etc.). In this study, as well as in all microcosm-based studies, the bottle effect may be one of the significant drivers for change, especially in the filtered fraction, reflected in proliferation of *Proteobacteria* and the decrease in abundance of *Firmicutes* and *Cyanobacteria* (Figs 3, S2 and S3).

## CONCLUSIONS

We detected metabolically active microorganisms residing in the <0.22 µm filtered river water fraction, where substrate utilization, CO_2_ production and biomass incorporation were observed in both radiolabelled and targeted metabolomics experiments. Although substrate depletion was very rapid in the unfiltered fraction, the 3-day lag in substrate use in the filterable fraction suggests it contains few microorganisms and/or that they are metabolically inactive. Considering the short residence time of freshwater in the Conwy catchment, we conclude that filterable microorganisms play a minor role in the processing of LMW DOC.

The most prominent phyla observed in microcosms across all samples were *Proteobacteria, Bacteroidetes, Actinobacteria, Firmicutes* and *Acidobacteria*. It should be noted that the filtered fraction contained many more *Firmicutes* and *Spirochetes* that are less active, as the community distribution shifted to containing more *Proteobacteria*, whereas the taxonomic groups in the total community remained largely unchanged, with the exception of *Actinobacteria, Armatimonadetes* and *Verrucomicrobia* that increased in numbers. It can be concluded that *Proteobacteria* were mainly responsible for the utilization of LMW DOC in the Conwy River within the filtered fraction and the community as a whole. As for Archaea, they were a minor constituent of the whole community, with *Euryarchaeota* dominant within all samples, and as the experiment progressed groups like *Crenarchaeota* and *Thaumarchaeota* increased within the 506 h period.

Although the makeup of the members within the microbial communities were not greatly altered by the addition of C substrates, the COG functional category of energy production and conversion showed changes, across the entire community, including the filterable fraction. The percentage of energy production/conversion COGs increased over the course of the 3 weeks in both fractions (i.e. gene expression was influenced by nanomolar concentrations of LMW DOC). Hence, it can be concluded that the bacteria and archaea residing in the river are generalists when it comes to the utilization of LMW DOC.

## Supplementary Material

fiaa244_Supplemental_Files
