# Supplementary material for: Utilization of low-molecular-weight organic compounds by the filterable fraction of a lotic microbiome
Source: FEMS Microbiol Ecol. 2020 Dec 2;97(2):fiaa244. doi: 10.1093/femsec/fiaa244 (PMC7864478; doi:10.1093/femsec/fiaa244)
Supplement: fiaa244_Supplemental_Files [file fiaa244_supplemental_files.zip › Supporting_material_utilization_of_LMW_DOC_draft6.docx]

**Supporting Material**

**Fig. S1 Residual concentrations of LMW upon incubation in filtered and unfiltered microcosms.** 16 metabolites: 11 amino acids (alanine, aspartic acid, glycine, isoleucine, leucine, phenylalanine, proline, serine, threonine, tyrosine, and valine), 3 carbohydrates (fructose, glucose, and sucrose), and two organic acids (citric acid and malic acid) (n=3) in both fractions in the filtered (blue) and unfiltered (red) were measured over the course of 506 h by GC MS.

**Fig. S2 Class level taxa of high-abundance OTUs in various treatments and substrate additions over 506 hours of incubation.** (A) filtered fraction and (B) total community (unfiltered fraction). The left column is the initial communities (n=3). The middle column shows the communities changing over the course of the three weeks w/o additional substrate versus the right column w/ additional substrates. Refer to Table S8 for further information regarding higher order taxa. Abundances lower than 50 individual gene counts were removed. See Methods section for more detail.

**Fig. S3 Family level taxa of high-abundance OTUs in various treatments and substrate additions over 506 hours of incubation.** (A) filtered fraction and (B) total community (unfiltered fraction). The left column is the initial communities (n=3). The middle column shows the communities changing over the course of the three weeks w/o additional substrate versus the right column w/ additional substrates. Refer to Table S8 for further information regarding higher order taxa. Abundances lower than 50 individual gene counts were removed. See Methods section for more detail.

**Table S1 Concentrations of amended substrates in microcosms**. The values for the unlabelled substrates are given in nM whereas labelled experiments are indicated in kBq/mL and nM. *NA indicates that the compound was not added

**Table S2 Mauchly’s test and Greenhouse-Geisser test for sphericity.** Mauchy’s W, p-value, and ε value are listed here. * Huyn-Feldt will need to be applied

**Table S3 Forward and reverse primers used for barcoded 16S rRNA gene amplicon sequencing.**

**Table S4 Quantification of LMW in microcosms.** Measurements were taken upon incubation of microcosms in both the unfiltered and filtered samples for each of the 16 amended compounds (n=3). Values indicated are relative peak areas of each compound based on GC-EI-QTOF-MS.

**Table S5 Absolute values of rates at various time intervals within both fractions.** The rate of ^14^C substrate depletion, ^14^CO_2_ production, and ^14^C Biomass incorporation across all three substrate types.

**Table S6 Summary of the t-testing for targeted metabolomics compounds.** Values were calculated from the relative abundance peak intensity of the GC system. Values for the initial 0 h and the final 506 h time point were used to check for utilization of each of the 16 compounds (n=3). P-values are reported where *indicates p <0.05, ** is p < 0.01, and ***is p <0.001.

**Table S7 Summary of the t-testing for COG distribution for both fractions and substrate addition.** Results use to determine the effects of experiment duration, treatment (Filtered vs unfiltered), and substrate addition on the distribution of COG functional categories. P-values are reported where * indicates p <0.05, ** is p < 0.01, and *** is p <0.001. NA denotes no value.

**Table S8 Overview of 16S rRNA gene amplicon sequencing.** Samples were labelled via their number and letter designations. Signal refers to the presence of a band of a 1.8% agarose gel. The number of reads retrieved per sample are also listed. *Negative controls. **Samples that produced no signal and were not included in subsequent analysis. † False positive result. NA (not applicable) indicates inability to sequence samples, even though there was a positive signal. ††Inability to sequence the sample, even with a positive signal after primers.

**Table S9 OTU taxonomic assignments in both fractions across all time points with and without substrate addition.** This table shows the taxonomic classification and absolute abundances of each OTU. Note: unassigned indicates that there is currently no name associated with that taxa level.

**Table S10 Overview of shotgun sequencing across initial (0 h), 141 h, and 506 h in both filtered fraction and unfiltered community with and without nutrient amendments.** Sample ID, time point taken, treatment (filtered vs unfiltered) and substrate addition (NA, +/). The number of contigs, coding sequences (CDS), annotations, and COG assignments. NA indicates that this is the initial community at the beginning of the experiment.
