# Supplementary material for: Utilization of low-molecular-weight organic compounds by the filterable fraction of a lotic microbiome
Source: FEMS Microbiol Ecol. 2020 Dec 2;97(2):fiaa244. doi: 10.1093/femsec/fiaa244 (PMC7864478; doi:10.1093/femsec/fiaa244)
Supplement: fiaa244_Supplemental_Files [file fiaa244_supplemental_files.zip › Table_S1_utilization_of_LMW_DOC_draft3.docx]

| ***Substrate*** | ***Unlabelled addition (nM)*** | ***Labelled addition (kBq/mL)*** | ***Labelled addition (nM)*** |
| --- | --- | --- | --- |
| Alanine | 90 | 0.008 | 14.22 |
| Arginine | 90 | 0.007 | 7.01 |
| Aspartic acid | 90 | 0.008 | 10.81 |
| Glutamic acid | 90 | 0.0125 | 13.15 |
| Glycine | 90 | 0.004 | 10.50 |
| Histidine | 90 | 0.0015 | 1.20 |
| Isoleucine | 90 | 0.005 | 4.21 |
| Leucine | 90 | 0.014 | 11.82 |
| Lysine | 90 | 0.006 | 5.04 |
| Phenylalanine | 90 | 0.008 | 4.07 |
| Proline | 90 | 0.005 | 4.84 |
| Serine | 90 | 0.004 | 6.67 |
| Threonine | 90 | 0.005 | 6.79 |
| Tyrosine | 90 | 0.004 | 2.13 |
| Valine | 90 | 0.008 | 9.61 |
| Lysine HCL | 90 | NA* | NA* |
| Methionine | 90 | NA* | NA* |
| Cystine | 90 | NA* | NA* |
| Glucose | 200 | 0.1 | 90.09 |
| Sucrose | 189 | 0.1 | 49.77 |
| Fructose | 200 | 0.1 | 448.21 |
| Formic acid | 200 | 0.1 | 523.78 |
| Malic acid | 200 | 0.1 | 523.78 |
| Citric acid | 200 | 0.1 | 232.19 |
