# Supplementary material for: Utilization of low-molecular-weight organic compounds by the filterable fraction of a lotic microbiome
Source: FEMS Microbiol Ecol. 2020 Dec 2;97(2):fiaa244. doi: 10.1093/femsec/fiaa244 (PMC7864478; doi:10.1093/femsec/fiaa244)
Supplement: fiaa244_Supplemental_Files [file fiaa244_supplemental_files.zip › Table_S10_utilization_of_LMW_DOC_draft1.docx]

| ***Sample ID*** | ***Time point*** | ***Treatment*** | ***Substrate addition*** | ***Contigs*** | ***CDS*** | ***Annotations*** | ***COG assignations*** |
| --- | --- | --- | --- | --- | --- | --- | --- |
| 29 | initial | Filtered | NA | 55448 | 65137 | 25084 | 24228 |
| 28L | 506 | Filtered | + | 82874 | 75794 | 26193 | 44711 |
| 31L | 506 | Filtered | - | 56595 | 63879 | 44306 | 44877 |
| 28i | 141 | Filtered | + | 46501 | 38146 | 18021 | 18296 |
| 31i | 141 | Filtered | - | 30881 | 17982 | 7946 | 8100 |
| U | initial | Unfiltered | NA | 77690 | 31847 | 14813 | 14841 |
| 36L | 506 | Unfiltered | + | 43038 | 24158 | 1191 | 893 |
| 37L | 506 | Unfiltered | - | 47339 | 18946 | 6936 | 6828 |
| 35i | 141 | Unfiltered | + | 71461 | 41921 | 21821 | 22401 |
| 38i | 141 | Unfiltered | - | 144906 | 85698 | 43310 | 46063 |
| 28 | initial | Filtered | NA | 74187 | 112132 | 38959 | 37031 |
| 29L | 506 | Filtered | + | 107976 | 80606 | 44657 | 45096 |
| 32L | 506 | Filtered | - | 82602 | 106232 | 77879 | 78659 |
| 29i | 141 | Filtered | + | 76921 | 79720 | 53277 | 54579 |
| 32i | 141 | Filtered | - | 68087 | 69763 | 45651 | 46643 |
| 34 | initial | Unfiltered | NA | 240978 | 122695 | 60376 | 60269 |
| 35L | 506 | Unfiltered | + | 25762 | 13018 | 2210 | 2142 |
| 39L | 506 | Unfiltered | - | 80895 | 32200 | 6559 | 6559 |
| 36i | 141 | Unfiltered | + | 256700 | 181140 | 97113 | 99402 |
| 37i | 141 | Unfiltered | - | 182931 | 107126 | 59150 | 60379 |
