# Supplementary material for: Utilization of low-molecular-weight organic compounds by the filterable fraction of a lotic microbiome
Source: FEMS Microbiol Ecol. 2020 Dec 2;97(2):fiaa244. doi: 10.1093/femsec/fiaa244 (PMC7864478; doi:10.1093/femsec/fiaa244)
Supplement: fiaa244_Supplemental_Files [file fiaa244_supplemental_files.zip › Table_S2_utilization_of_LMW_DOC_draft3.docx]

|  |  |  |  | ***ε value*** | |
| --- | --- | --- | --- | --- | --- |
| ***Measurement*** | ***Substrate*** | ***Mauchly's W*** | ***p-value*** | ***Greenhouse-Geisser*** | ***Huynh-Feldt*** |
| ^14^C Substrate Depletion | ^14^C Amino acids | 0.614 | 0.003 | 0.722 | 1.000 |
| ^14^C Substrate Depletion | ^14^C Organic acids | 0.301 | <0.001 | 0.589 | 0.959 |
| ^14^C Substrate Depletion | ^14^C Sugars | 0.137 | <0.001 | 0.537 | 0.800 |
| ^14^CO_2_ production | ^14^C Amino acids | 0.773* | 0.045 | 0.815 | 1.000 |
| ^14^CO_2_ production | ^14^C Organic acids | 0.222 | <0.001 | 0.562 | 0.895 |
| ^14^CO_2_ production | ^14^C Sugars | 0.519 | <0.001 | 0.675 | 1.000 |
| ^14^C Biomass incorporation | ^14^C Amino acids | 0.913* | 0.336 | 0.920 | 1.000 |
| ^14^C Biomass incorporation | ^14^C Organic acids | 0.559 | 0.003 | 0.694 | 1.000 |
| ^14^C Biomass incorporation | ^14^C Sugars | 0.141 | <0.001 | 0.538 | 0.802 |
