# Supplementary material for: Utilization of low-molecular-weight organic compounds by the filterable fraction of a lotic microbiome
Source: FEMS Microbiol Ecol. 2020 Dec 2;97(2):fiaa244. doi: 10.1093/femsec/fiaa244 (PMC7864478; doi:10.1093/femsec/fiaa244)
Supplement: fiaa244_Supplemental_Files [file fiaa244_supplemental_files.zip › Table_S3_utilization_of_LMW_DOC_draft3.docx]

| ***Primer name*** | ***Sequence (5’ to 3’)*** |
| --- | --- |
| F515DI2 | CAAGCAGAAGACGGCATACGAGATGTGACTGGAGTTCAGACGTGTGCTCTTCCGATCT TGCAGATCCAAC GTGBCAGCMGCCGCGGTAA |
| F515DI3 | CAAGCAGAAGACGGCATACGAGATGTGACTGGAGTTCAGACGTGTGCTCTTCCGATCT CCATCACATAGG GTGBCAGCMGCCGCGGTAA |
| F515DI4 | CAAGCAGAAGACGGCATACGAGATGTGACTGGAGTTCAGACGTGTGCTCTTCCGATCT GTGGTATGGGAG T GTGBCAGCMGCCGCGGTAA |
| F515DI5 | CAAGCAGAAGACGGCATACGAGATGTGACTGGAGTTCAGACGTGTGCTCTTCCGATCT ACTTTAAGGGTG T GTGBCAGCMGCCGCGGTAA |
| F515DI6 | CAAGCAGAAGACGGCATACGAGATGTGACTGGAGTTCAGACGTGTGCTCTTCCGATCT GAGCAACATCCT T GTGBCAGCMGCCGCGGTAA |
| F515DI7 | CAAGCAGAAGACGGCATACGAGATGTGACTGGAGTTCAGACGTGTGCTCTTCCGATCT TGTTGCGTTTCT GT GTGBCAGCMGCCGCGGTAA |
| F515DI8 | CAAGCAGAAGACGGCATACGAGATGTGACTGGAGTTCAGACGTGTGCTCTTCCGATCT ATGTCCGACCAA GT GTGBCAGCMGCCGCGGTAA |
| F515DI9 | CAAGCAGAAGACGGCATACGAGATGTGACTGGAGTTCAGACGTGTGCTCTTCCGATCT AGGTACGCAATT GT GTGBCAGCMGCCGCGGTAA |
| F515DI10 | CAAGCAGAAGACGGCATACGAGATGTGACTGGAGTTCAGACGTGTGCTCTTCCGATCT ACAGCCACCCAT CGA GTGBCAGCMGCCGCGGTAA |
| F515DI11 | CAAGCAGAAGACGGCATACGAGATGTGACTGGAGTTCAGACGTGTGCTCTTCCGATCT TGTCTCGCAAGC CGA GTGBCAGCMGCCGCGGTAA |
| F515DI12 | CAAGCAGAAGACGGCATACGAGATGTGACTGGAGTTCAGACGTGTGCTCTTCCGATCT GAGGAGTAAAGC CGA GTGBCAGCMGCCGCGGTAA |
| F515DI13 | CAAGCAGAAGACGGCATACGAGATGTGACTGGAGTTCAGACGTGTGCTCTTCCGATCT GTTACGTGGTTG ATGA GTGBCAGCMGCCGCGGTAA |
| F515DI14 | CAAGCAGAAGACGGCATACGAGATGTGACTGGAGTTCAGACGTGTGCTCTTCCGATCT TACCGCCTCGGA ATGA GTGBCAGCMGCCGCGGTAA |
| F515DI15 | CAAGCAGAAGACGGCATACGAGATGTGACTGGAGTTCAGACGTGTGCTCTTCCGATCT CGTAAGATGCCT ATGA GTGBCAGCMGCCGCGGTAA |
| F515DI16 | CAAGCAGAAGACGGCATACGAGATGTGACTGGAGTTCAGACGTGTGCTCTTCCGATCT TACCGGCTTGCA TGCGA GTGBCAGCMGCCGCGGTAA |
| F515DI17 | CAAGCAGAAGACGGCATACGAGATGTGACTGGAGTTCAGACGTGTGCTCTTCCGATCT ATCTAGTGGCAA TGCGA GTGBCAGCMGCCGCGGTAA |
| F515DI18 | CAAGCAGAAGACGGCATACGAGATGTGACTGGAGTTCAGACGTGTGCTCTTCCGATCT CCAGGGACTTCT TGCGT GTGBCAGCMGCCGCGGTAA |
| F515DI19 | CAAGCAGAAGACGGCATACGAGATGTGACTGGAGTTCAGACGTGTGCTCTTCCGATCT CACCTTACCTTA GAGTGG GTGBCAGCMGCCGCGGTAA |
| F515DI24 | CAAGCAGAAGACGGCATACGAGATGTGACTGGAGTTCAGACGTGTGCTCTTCCGATCT GAGACTATATGC CCTGGAG GTGBCAGCMGCCGCGGTAA |
| R806DI2 | AATGATACGGCGACCACCGAGATCTACACTCTTTCCCTACACGACGCTCTTCCGATCT TGCAGATCCAAC GGACTACHVGGGTWTCTAAT |
| R806DI3 | AATGATACGGCGACCACCGAGATCTACACTCTTTCCCTACACGACGCTCTTCCGATCT CCATCACATAGG GGACTACHVGGGTWTCTAAT |
| R806DI4 | AATGATACGGCGACCACCGAGATCTACACTCTTTCCCTACACGACGCTCTTCCGATCT GTGGTATGGGAG A GGACTACHVGGGTWTCTAAT |
| R806DI5 | AATGATACGGCGACCACCGAGATCTACACTCTTTCCCTACACGACGCTCTTCCGATCT ACTTTAAGGGTG A GGACTACHVGGGTWTCTAAT |
| R806DI6 | AATGATACGGCGACCACCGAGATCTACACTCTTTCCCTACACGACGCTCTTCCGATCT GAGCAACATCCT A GGACTACHVGGGTWTCTAAT |
