# Supplementary material for: Utilization of low-molecular-weight organic compounds by the filterable fraction of a lotic microbiome
Source: FEMS Microbiol Ecol. 2020 Dec 2;97(2):fiaa244. doi: 10.1093/femsec/fiaa244 (PMC7864478; doi:10.1093/femsec/fiaa244)
Supplement: fiaa244_Supplemental_Files [file fiaa244_supplemental_files.zip › Table_S5_utilization_of_LMW_DOC_draft3.docx]

|  | ***Unfiltered*** | | |  |  |  |  |  |  |
| --- | --- | --- | --- | --- | --- | --- | --- | --- | --- |
|  | ***^14^C Substrate Depletion*** | | | ***^14^CO_2_ production*** | | | ***^14^C Biomass incorporation*** | | |
| ***Time interval*** | ***Amino acids*** | ***Organic acids*** | ***Sugars*** | ***Amino acids*** | ***Organic acids*** | ***Sugars*** | ***Amino acids*** | ***Organic acids*** | ***Sugars*** |
| 0-6 h | 0.16 | 0.18 | 0.18 | 0.06 | 0.07 | 0.05 | 0.11 | 0.11 | 0.13 |
| 0-22 h | 3.28 | 3.45 | 3.77 | 0.20 | 0.41 | 0.09 | 3.08 | 3.04 | 3.68 |
| 0-49 h | 1.52 | 1.85 | 1.81 | 0.15 | 0.37 | 0.11 | 1.37 | 1.47 | 1.70 |
| 0-74 h | 1.03 | 1.23 | 1.18 | 0.13 | 0.30 | 0.13 | 0.90 | 0.93 | 1.05 |
| 0-141 h | 0.53 | 0.64 | 0.59 | 0.12 | 0.21 | 0.11 | 0.41 | 0.43 | 0.48 |
| 0-333 h | 0.24 | 0.27 | 0.26 | 0.08 | 0.10 | 0.07 | 0.16 | 0.17 | 0.19 |
| 0-506 h | 0.16 | 0.18 | 0.18 | 0.06 | 0.07 | 0.05 | 0.11 | 0.11 | 0.13 |
| 22-506 h | 0.02 | 0.03 | 0.01 | 0.05 | 0.06 | 0.05 | 0.03 | 0.03 | 0.03 |
| 49-506 h | 0.02 | 0.00 | 0.00 | 0.05 | 0.04 | 0.04 | 0.03 | 0.04 | 0.04 |
| 74-506 h | 0.01 | 0.00 | 0.00 | 0.04 | 0.04 | 0.04 | 0.03 | 0.03 | 0.03 |
| 141-506 h | 0.02 | 0.00 | 0.02 | 0.03 | 0.02 | 0.03 | 0.01 | 0.02 | 0.01 |
|  |  |  |  |  |  |  |  |  |  |
|  |  |  |  |  |  |  |  |  |  |
|  | ***Filtered*** | | |  |  |  |  |  |  |
|  | ***^14^C Substrate Depletion*** | | | ***^14^CO2 production*** | | | ***^14^C Biomass incorporation*** | | |
| ***Time interval*** | ***Amino acids*** | ***Organic acids*** | ***Sugars*** | ***Amino acids*** | ***Organic acids*** | ***Sugars*** | ***Amino acids*** | ***Organic acids*** | ***Sugars*** |
| 0-6 h | 0.14 | 0.11 | 0.17 | 0.03 | 0.04 | 0.04 | 0.11 | 0.08 | 0.12 |
| 0-22 h | 1.31 | 1.00 | 0.63 | 0.00 | 0.02 | 0.00 | 1.31 | 1.02 | 0.63 |
| 0-49 h | 0.45 | 0.29 | 0.27 | 0.00 | 0.01 | 0.00 | 0.47 | 0.30 | 0.27 |
| 0-74 h | 0.07 | 0.40 | 0.03 | 0.01 | 0.03 | 0.00 | 0.18 | 0.56 | 0.13 |
| 0-141 h | 0.48 | 0.33 | 0.60 | 0.03 | 0.05 | 0.05 | 0.45 | 0.38 | 0.55 |
| 0-333 h | 0.21 | 0.16 | 0.25 | 0.04 | 0.05 | 0.05 | 0.18 | 0.15 | 0.20 |
| 0-506 h | 0.14 | 0.11 | 0.17 | 0.03 | 0.04 | 0.04 | 0.11 | 0.08 | 0.12 |
| 22-506 h | 0.20 | 0.16 | 0.20 | 0.03 | 0.04 | 0.05 | 0.06 | 0.04 | 0.10 |
| 49-506 h | 0.20 | 0.15 | 0.22 | 0.03 | 0.05 | 0.05 | 0.07 | 0.06 | 0.11 |
| 74-506 h | 0.15 | 0.06 | 0.19 | 0.03 | 0.04 | 0.05 | 0.10 | 0.00 | 0.12 |
| 141-506 h | 0.01 | 0.03 | 0.00 | 0.03 | 0.04 | 0.04 | 0.02 | 0.03 | 0.04 |
