# Supplementary material for: Utilization of low-molecular-weight organic compounds by the filterable fraction of a lotic microbiome
Source: FEMS Microbiol Ecol. 2020 Dec 2;97(2):fiaa244. doi: 10.1093/femsec/fiaa244 (PMC7864478; doi:10.1093/femsec/fiaa244)
Supplement: fiaa244_Supplemental_Files [file fiaa244_supplemental_files.zip › Table_S6_utilization_of_LMW_DOC_draft3.docx]

|  | ***Filtered*** | ***Unfiltered*** |
| --- | --- | --- |
| ***Compound*** | ***p-value*** | ***p-value*** |
| Alanine | 0.01* | 0.075 |
| Aspartic acid | 0.007** | 0.244 |
| Glycine | 0.006** | < 0.001*** |
| Isoleucine | 0.001** | 0.008** |
| Leucine | 0.001** | 0.075 |
| Phenylalanine | 0.001** | 0.012* |
| Proline | 0.004** | 0.033* |
| Serine | 0.005** | 0.044* |
| Threonine | 0*** | 0.046* |
| Tyrosine | 0.012* | 0.004** |
| Valine | 0.003** | 0.015* |
| Fructose | < 0.001*** | 0.12 |
| Glucose | 0.001** | 0.109 |
| Sucrose | 0*** | 0.147 |
| Citric acid | < 0.001*** | < 0.001*** |
| Malic acid | < 0.001*** | 0.006** |
