# Supplementary material for: Utilization of low-molecular-weight organic compounds by the filterable fraction of a lotic microbiome
Source: FEMS Microbiol Ecol. 2020 Dec 2;97(2):fiaa244. doi: 10.1093/femsec/fiaa244 (PMC7864478; doi:10.1093/femsec/fiaa244)
Supplement: fiaa244_Supplemental_Files [file fiaa244_supplemental_files.zip › Table_S7_utilization_of_LMW_DOC_draft3.docx]

|  | ***Filtered*** |  | ***Unfiltered*** |  |
| --- | --- | --- | --- | --- |
| ***COG Category*** | ***w/ substrates*** | ***w/o substrates*** | ***w/ substrates*** | ***w/o substrates*** |
| Energy production and conversion | 0.416 | 0.179 | 0.864 | 0.253 |
| Cell cycle control, cell division, chromosome partitioning | 0.221 | 0.281 | 0.356 | 0.218 |
| Amino acid transport and metabolism | 0.047* | 0.019* | 0.125 | 0.912 |
| Nucleotide transport and metabolism | 0.182 | 0.017* | 0.153 | 0.394 |
| Carbohydrate transport and metabolism | 0.698 | 0.327 | 0.262 | 0.364 |
| Coenzyme transport and metabolism | 0.015* | 0.025* | 0.807 | 0.551 |
| Lipid transport and metabolism | 0.121 | 0.060 | 0.233 | 0.912 |
| Translation, ribosomal structure and biogenesis | 0.003** | 0.005** | 0.918 | 0.576 |
| Transcription | 0.012* | 0.009** | 0.412 | 0.398 |
| Replication, recombination and repair | 0.365 | 0.218 | 0.421 | 0.324 |
| Cell wall/membrane/envelope biogenesis | 0.020* | 0.061 | 0.823 | 0.155 |
| Cell motility | 0.029* | 0.018* | 0.869 | 0.958 |
| Posttranslational modification, protein turnover, chaperones | 0.011* | 0.056 | 0.041* | 0.408 |
| Inorganic ion transport and metabolism | 0.001*** | 0.004** | 0.702 | 0.529 |
| Secondary metabolites biosynthesis, transport and catabolism | 0.010** | 0.103 | 0.135 | 0.443 |
| Signal transduction mechanisms | 0.036* | 0.004** | 0.589 | 0.313 |
| Intracellular trafficking, secretion, and vesicular transport | 0.463 | 0.062 | 0.388 | 0.546 |
| Defense mechanisms | NA | NA | NA | NA |
| RNA processing and modification | 0.115 | 0.054 | 0.117 | 0.598 |
| Chromatin structure and dynamics | 0.506 | 0.190 | 0.691 | 0.695 |
| General function prediction only | 0.599 | 0.997 | 0.058 | 0.403 |
| Extracellular structures | 0.125 | 0.122 | 0.481 | 0.305 |
| Mobilome: prophages, transposons | NA | NA | NA | NA |
| Nuclear structure | NA | NA | NA | NA |
| Cytoskeleton | 0.350 | 0.018* | 0.286 | 0.053 |
| Function unknown | 0.040* | 0.037* | 0.517 | 0.566 |
