# Supplementary material for: Utilization of low-molecular-weight organic compounds by the filterable fraction of a lotic microbiome
Source: FEMS Microbiol Ecol. 2020 Dec 2;97(2):fiaa244. doi: 10.1093/femsec/fiaa244 (PMC7864478; doi:10.1093/femsec/fiaa244)
Supplement: fiaa244_Supplemental_Files [file fiaa244_supplemental_files.zip › Table_S8_utilization_of_LMW_DOC_draft3.docx]

| ***Sample ID*** | ***Hours after addition*** | ***Treatment*** | ***Substrate Addition*** | ***Signal*** | ***Meta-barcode ID*** | ***Number of Reads*** |
| --- | --- | --- | --- | --- | --- | --- |
| 28 | 0 | Filtered | + | + | Bangor.L.28.NA | 241 |
| 29 | 0 | Filtered | + | + | Bangor.L.29.NA | 577 |
| 30 | 0 | Filtered | + | + | Bangor.L.30.NA | 560 |
| 34 | 0 | Unfiltered | + | + | Bangor.L.34.NA | 10,161 |
| 35 | 0 | Unfiltered | + | + | Bangor.L.35.NA | 5,558 |
| 36 | 0 | Unfiltered | + | + | Bangor.L.36.NA | 6,963 |
| 40*† | 0 | Blank | + | + | NA†† | NA |
| 41* | 0 | Blank | + | - |  | NA |
| 42* | 0 | Blank | + | - |  | NA |
| 28g** | 49 | Filtered | + | - |  | NA |
| 29g | 49 | Filtered | + | + | Bangor.L.29.g | 5 |
| 30g | 49 | Filtered | + | + | Bangor.L.30.g | 909 |
| 31g | 49 | Filtered | - | + | Bangor.L.31.g | 782 |
| 32g** | 49 | Filtered | - | - |  | NA |
| 33g | 49 | Filtered | - | + | Bangor.L.33.g | 1,755 |
| 34g | 49 | Unfiltered | + | + | Bangor.L.34.g | 18,361 |
| 35g | 49 | Unfiltered | + | + | Bangor.L.35.g | 17,286 |
| 36g | 49 | Unfiltered | + | + | Bangor.L.36.g | 11,079 |
| 37g | 49 | Unfiltered | - | + | Bangor.L.37.g | 37,037 |
| 38g | 49 | Unfiltered | - | + | Bangor.L.38.g | 33,406 |
| 39g | 49 | Unfiltered | - | + | Bangor.L.39.g | 25,473 |
| 28i | 141 | Filtered | + | + | Bangor.L.28.i | 19,519 |
| 29i | 141 | Filtered | + | + | Bangor.L.29.i | 18,969 |
| 30i | 141 | Filtered | + | + | Bangor.L.30.i | 11,165 |
| 31i | 141 | Filtered | - | + | Bangor.L.31.i | 16,445 |
| 32i | 141 | Filtered | - | + | Bangor.L.32.i | 28,145 |
| 33i | 141 | Filtered | - | + | Bangor.L.33.i | 26,989 |
| 34i | 141 | Unfiltered | + | + | Bangor.L.34.i | 19,473 |
| 35i | 141 | Unfiltered | + | + | Bangor.L.35.i | 18,626 |
| 36i | 141 | Unfiltered | + | + | Bangor.L.36.i | 18,241 |
| 37i** | 141 | Unfiltered | - | - |  | NA |
| 38i | 141 | Unfiltered | - | + | Bangor.L.38.i | 15,668 |
| 39i | 141 | Unfiltered | - | + | Bangor.L.39.i | 18,883 |
| 28k | 333 | Filtered | + | + | Bangor.L.28.k | 24,025 |
| 29k | 333 | Filtered | + | + | Bangor.L.29.k | 16,516 |
| 30k | 333 | Filtered | + | + | Bangor.L.30.k | 24,453 |
| 31k | 333 | Filtered | - | + | Bangor.L.31.k | 24,763 |
| 32k | 333 | Filtered | - | + | Bangor.L.32.k | 27,545 |
| 33k | 333 | Filtered | - | + | Bangor.L.33.k | 17,912 |
| 34k | 333 | Unfiltered | + | + | Bangor.L.34.k | 13,228 |
| 35k | 333 | Unfiltered | + | + | Bangor.L.35.k | 13,275 |
| 36k | 333 | Unfiltered | + | + | Bangor.L.36.k | 12,981 |
| 37k | 333 | Unfiltered | - | + | Bangor.L.37.k | 18,043 |
| 38k | 333 | Unfiltered | - | + | Bangor.L.38.k | 16,158 |
| 39k | 333 | Unfiltered | - | + | Bangor.L.39.k | 14,267 |
| 28L | 506 | Filtered | + | + | Bangor.L.28.L | 4,096 |
| 29L | 506 | Filtered | + | + | Bangor.L.29.L | 4,906 |
| 30L | 506 | Filtered | + | + | Bangor.L.30.L | 9,605 |
| 31L | 506 | Filtered | - | + | Bangor.L.31.L | 10,439 |
| 32L | 506 | Filtered | - | + | Bangor.L.32.L | 6,882 |
| 33L | 506 | Filtered | - | + | Bangor.L.33.L | 9,041 |
| 34L | 506 | Unfiltered | + | + | Bangor.L.34.L | 1,380 |
| 35L | 506 | Unfiltered | + | + | Bangor.L.35.L | 5,071 |
| 36L | 506 | Unfiltered | + | + | Bangor.L.36.L | 2,927 |
| 37L | 506 | Unfiltered | - | + | NA†† | NA |
| 38L | 506 | Unfiltered | - | + | Bangor.L.38.L | 3,261 |
| 39L | 506 | Unfiltered | - | + | Bangor.L.39.L | 5,355 |
| 40L* | 506 | Blank | + | - |  | NA |
| 41L* | 506 | Blank | + | - |  | NA |
| 42L* | 506 | Blank | + | - |  | NA |
