# Supplementary figures and images for: Utilization of low-molecular-weight organic compounds by the filterable fraction of a lotic microbiome
Source: FEMS Microbiol Ecol. 2020 Dec 2;97(2):fiaa244. doi: 10.1093/femsec/fiaa244 (PMC7864478; doi:10.1093/femsec/fiaa244)

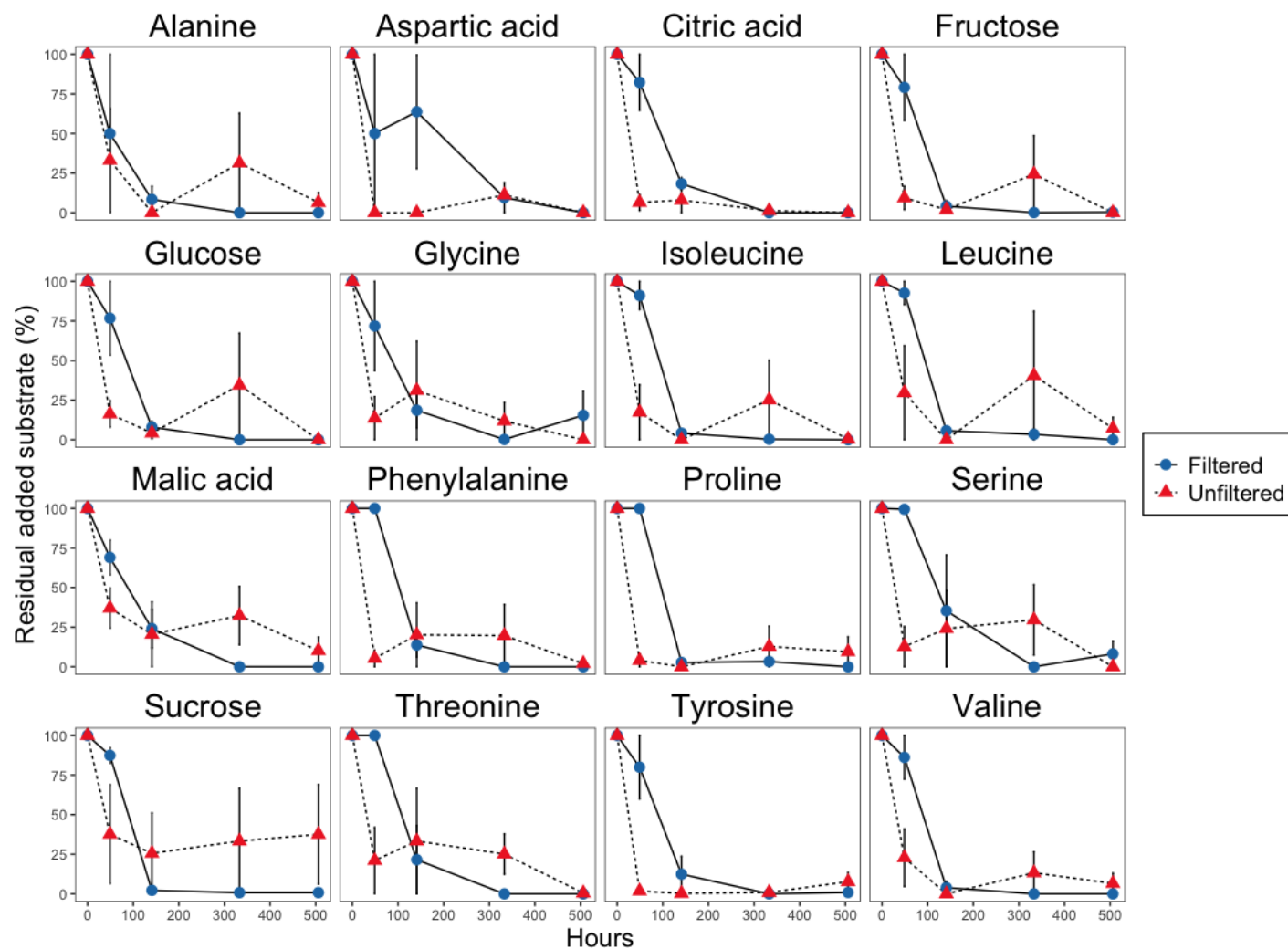

Supplement: fiaa244_Supplemental_Files [file fiaa244_supplemental_files.zip › Figure_S1_utilization_of_LMW_DOC.pdf]

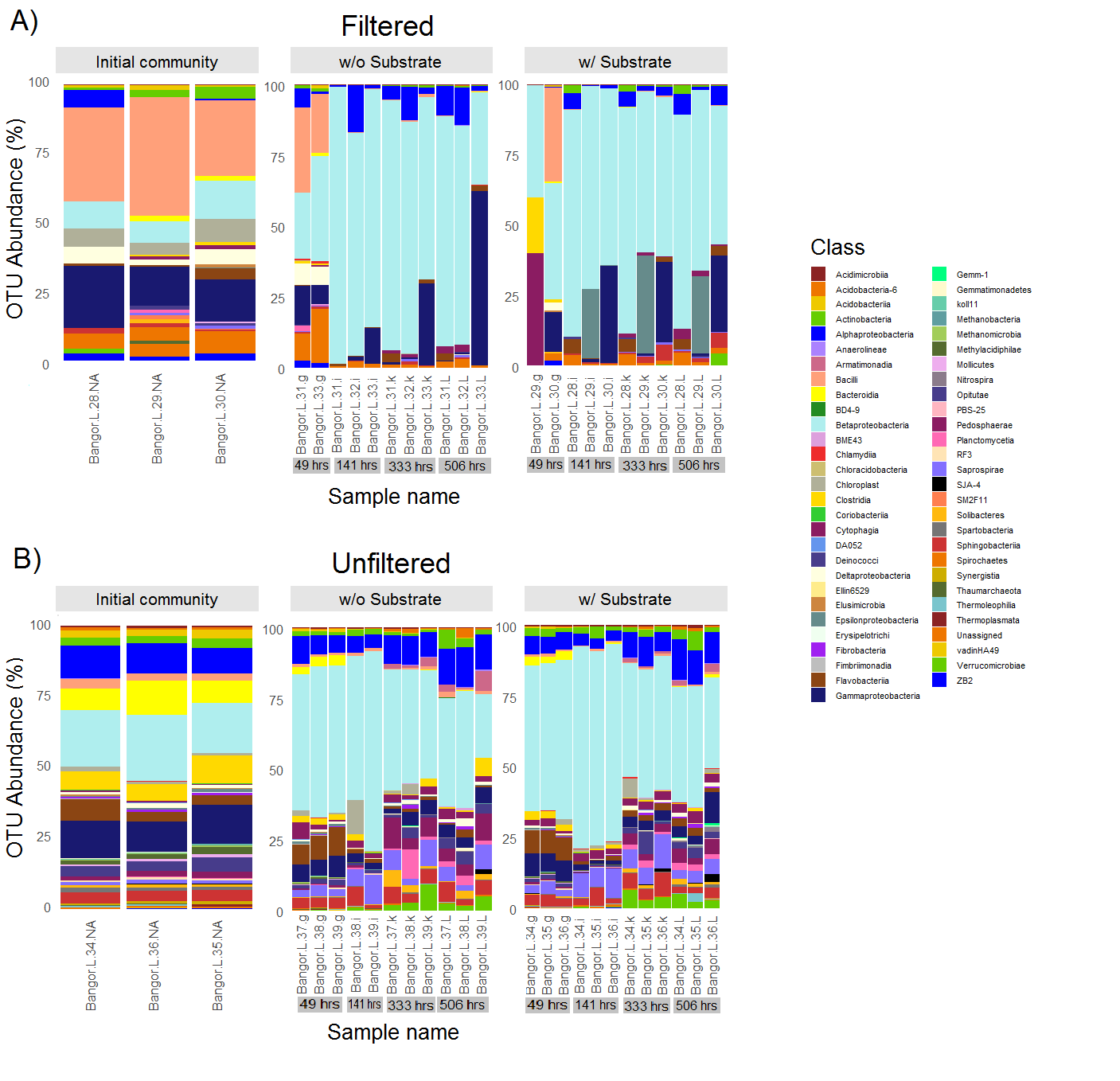

Supplement: fiaa244_Supplemental_Files [file fiaa244_supplemental_files.zip › Figure_S2_utilization_of_LMW_DOC_draft2.png]

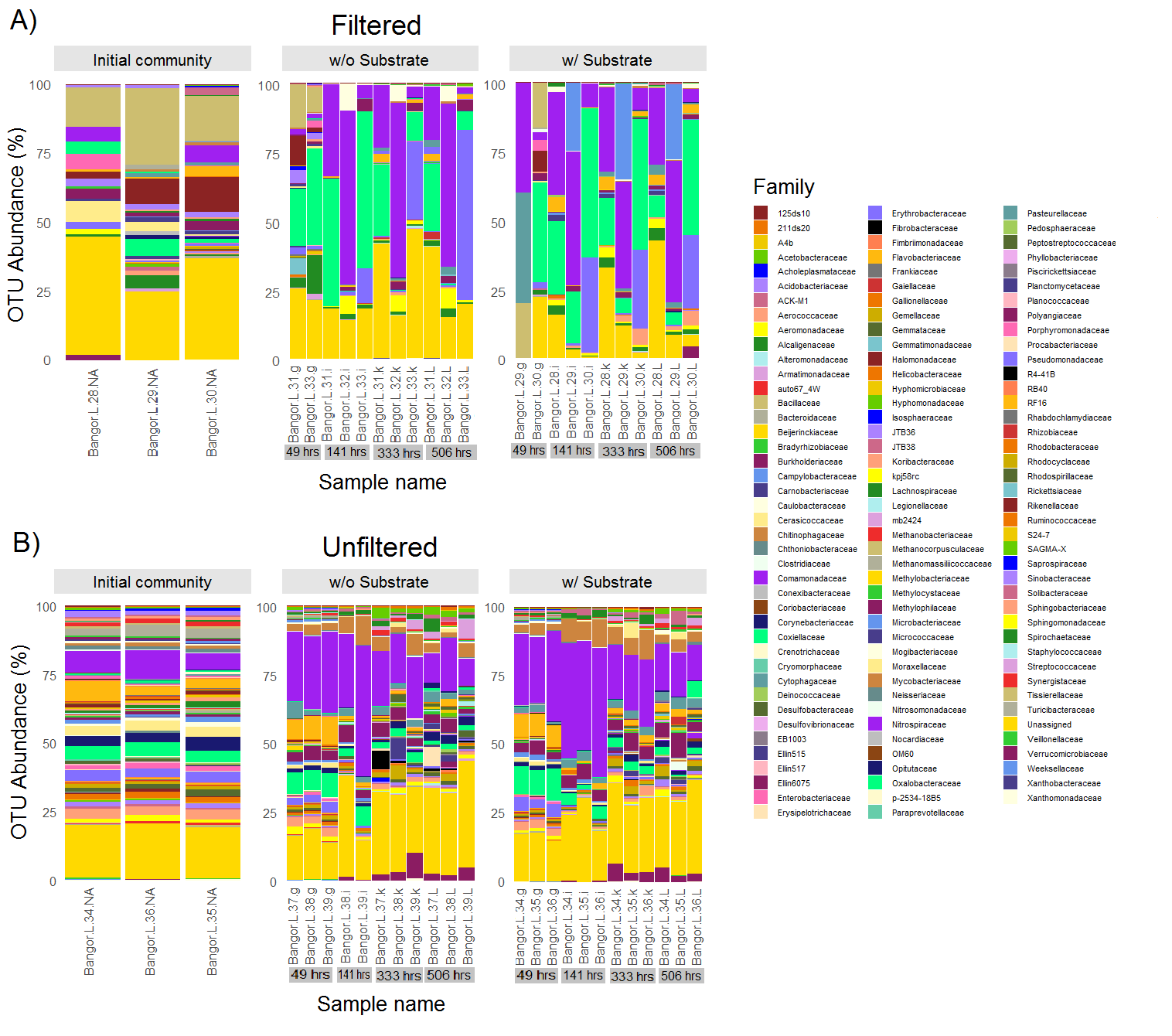

Supplement: fiaa244_Supplemental_Files [file fiaa244_supplemental_files.zip › Figure_S3_utilization_of_LMW_DOC_draft1.png]
